# Single-Dose Intrathecal Dorsal Root Ganglia Toxicity of Onasemnogene Abeparvovec in Cynomolgus Monkeys

**DOI:** 10.1089/hum.2021.255

**Published:** 2022-07-13

**Authors:** Francis Fonyuy Tukov, Keith Mansfield, Mark Milton, Emily Meseck, Kelley Penraat, Deepa Chand, Andreas Hartmann

**Affiliations:** ^1^ Department of Preclinical Safety/Translational Medicine, Novartis Pharmaceuticals Corporation, East Hanover, New Jersey, USA.; ^2^ Department of Discovery and Investigative Pathology, Novartis Institutes for BioMedical Research, Cambridge, Massachusetts, USA.; ^3^ Department of PK Sciences—Gene Therapy TA, Novartis Institutes for BioMedical Research, Cambridge, Massachusetts, USA.; ^4^ Novartis Pharmaceuticals Corporation, East Hanover, New Jersey, USA.; ^5^ Novartis Institutes for BioMedical Research, Cambridge, Massachusetts, USA.; ^6^ Novartis Gene Therapies, Inc., Bannockburn, Illinois, USA.; ^7^ Department of Pediatrics, Washington University School of Medicine and St Louis Children's Hospital, St Louis, Missouri, USA.; ^8^ Department of Preclinical Safety, Novartis Pharma AG, Basel, Switzerland.

**Keywords:** adeno-associated viruses, dorsal root ganglion, intrathecal administration, nonhuman primate, onasemnogene abeparvovec, spinal muscular atrophy

## Abstract

Intravenous onasemnogene abeparvovec is approved for the treatment of spinal muscular atrophy in children < 2 years. For later-onset patients, intrathecal onasemnogene abeparvovec may be advantageous over intravenous administration. Recently, microscopic dorsal root ganglion (DRG) changes were observed in nonhuman primates (NHPs) following intrathecal onasemnogene abeparvovec administration. To characterize these DRG findings, two NHP studies evaluating intrathecal onasemnogene abeparvovec administration were conducted: a 12-month study with a 6-week interim cohort and a 13-week study with a 2-week interim cohort. The latter investigated the potential impact of prednisolone or rituximab plus everolimus on DRG toxicity. An additional 6-month, single-dose, intravenous NHP study conducted in parallel evaluated onasemnogene abeparvovec safety (including DRG toxicity) with or without prednisolone coadministration. Intrathecal onasemnogene abeparvovec administration was well tolerated and not associated with clinical observations. Microscopic onasemnogene abeparvovec-related changes were observed in the DRG and trigeminal ganglion (TG) and included mononuclear cell inflammation and/or neuronal degeneration, which was colocalized with high vector transcript expression at 6 weeks postdose. Incidence and severity of DRG changes were generally decreased after 52 weeks compared with 6 weeks postdose. Other onasemnogene abeparvovec-related microscopic findings of axonal degeneration, mononuclear cell infiltrates and/or gliosis in the spinal cord, dorsal spinal nerve root/spinal nerves, and/or peripheral nerves were absent or found at decreased incidences and/or severities after 52 weeks. DRG and/or TG microscopic findings following intravenous onasemnogene abeparvovec dosing included minimal to slight neuronal degeneration and mononuclear cell inflammation at 6 weeks and 6 months postdose. Nervous system microscopic findings following intrathecal onasemnogene abeparvovec (≥1.2 × 10^13^ vg/animal) trended toward resolution after 52 weeks, supporting nonprogression of changes, including in the DRG. Onasemnogene abeparvovec-related DRG findings were not associated with electrophysiology changes and were not ameliorated by prednisolone or rituximab plus everolimus coadministration. The pathogenesis is possibly a consequence of increased vector genome transduction and/or transgene expression.

## INTRODUCTION

Onasemnogene abeparvovec is an approved one-time intravenous infusion therapy indicated for the treatment of patients with 5q spinal muscular atrophy (SMA) with a biallelic mutation in the *survival motor neuron 1* (*SMN1*) gene and a clinical diagnosis of SMA type 1, or patients with 5q SMA with a biallelic mutation in the *SMN1* gene and up to three copies of the *SMN2* gene. SMA is an autosomal recessive, neurodegenerative disease resulting from biallelic *SMN1* gene mutations that causes a survival motor neuron (SMN) protein deficiency, leading to motor neuron degeneration and early mortality.^1^ SMA is classified into four phenotypes based on age of onset and greatest motor function achieved.

Type 0 represents severe, antenatal-onset SMA, which is not compatible with life.^2,3^ SMA type 1 patients present symptomatically within the first 6 months of life, never attain independent sitting, and die or require permanent ventilation before 2 years of age, if untreated.^4–6^ SMA type 2 manifests within 18 months of age, and patients are able to sit unassisted but never walk independently. These patients experience significant cumulative morbidity related to worsening contractures, spinal curvature, muscle atrophy, and slow but progressive motor function loss over time.^7^ SMA type 2 patients have a life expectancy of 20–40 years. SMA type 3 patients attain the ability to walk unaided. SMA type 4 is an adult-onset form.

Nonclinical studies have consistently demonstrated that intravenous or intrathecal administration of self-complementary adeno-associated virus serotype 9 (scAAV9)-SMN or scAAV9–green fluorescent protein targets spinal motor neurons.^8–10^ Furthermore, intravenous or intrathecal delivery of functional *SMN* via AAV gene therapy can abrogate the SMA phenotype in mouse models of disease.^11–14^

Onasemnogene abeparvovec is a recombinant biological product that comprised a nonreplicating, recombinant, scAAV9 capsid shell containing a functional copy of the human *SMN* gene. The *SMN* gene is under the control of the cytomegalovirus enhancer/chicken-β-actin hybrid promoter as well as two AAV inverted terminal repeats derived from AAV2. Nonclinical studies, predominantly conducted in murine species, provided the foundation for the development of onasemnogene abeparvovec, the first approved systemic gene therapy administered as a single dose via intravenous infusion. Intravenous onasemnogene abeparvovec in patients is well tolerated, with an adverse event profile that is monitorable and largely manageable.^15^

The intrathecal route of onasemnogene abeparvovec administration may be the preferred approach for treatment of later-onset SMA patients. This approach reduces the dose in the central nervous system (CNS) by ∼10-fold relative to the approved intravenous dose and results in onasemnogene abeparvovec distribution throughout the spinal cord. In addition, intrathecal administration reduces the viral vector distribution in non-CNS tissues (*e.g.,* liver) and may result in an improved systemic safety profile.^9,10,16^

In a preliminary 2-week biodistribution study with cynomolgus monkeys (nonhuman primates [NHPs]) to support the development of intrathecal onasemnogene abeparvovec administration, an unexpected change was observed in the dorsal root ganglion (DRG) of onasemnogene abeparvovec–treated NHPs using a dose of 3 × 10^13^ vg/animal given with or without iohexol-based contrast agents. The findings resulted in a US Food and Drug Administration (FDA) partial clinical hold for clinical trials of intrathecal onasemnogene abeparvovec administration.

Results from this NHP study demonstrated microscopic changes in the DRG, including mononuclear cell inflammatory infiltrate that was occasionally accompanied by neuronal satellitosis or neuronal necrosis at mild to marked severity.^15^ These findings were asymptomatic and unassociated with *in vivo* clinical observations.^15^

Data from this 2-week study^15^ were not sufficient to characterize the progression of the observed DRG toxicity and evaluate long-term safety of intrathecally administered onasemnogene abeparvovec. Therefore, to characterize the finding further and investigate potential underlying mechanisms causing DRG changes, two nonclinical toxicity studies were conducted in NHPs administered onasemnogene abeparvovec by the intrathecal route of administration. The first intrathecal study was a 12-month, single-dose, good laboratory practice (GLP)‒compliant toxicity study with a 6-week interim cohort at dose concentrations of 1.2 × 10^13^, 3.0 × 10^13^, or 6.0 × 10^13^ vg/animal to explore dose response, characterize microscopic DRG findings, and assess the extent of any related or secondary microscopic findings in the nervous system of NHPs.

The doses selected for this study were based on the anticipated clinical dose. The low dose was the anticipated clinical dose, and the mid and high doses were 2.5 × and 5 × the anticipated clinical dose given the narrow safety margin of gene therapy products. This study also evaluated safety of intrathecal administration, assessing the persistence or reversibility of any effects as well as the progression or resolution of any nervous system findings. The second study was a 13-week, single-dose, mechanistic, non-GLP toxicity study with a 2-week interim cohort at a dose of 3.0 × 10^13^ vg/animal. This study investigated whether the microscopic DRG findings following intrathecal onasemnogene abeparvovec administration could be prevented or mitigated by coadministration of prednisolone or rituximab plus everolimus.

These pharmacologic agents were selected for their anti-inflammatory (reduction of chemokine, cytokine, and other inflammatory-mediated events related to innate and adaptive immunity) and/or immunosuppressive (reduction of B and T cell expansion and/or depletion) properties in NHPs at dose concentrations that would not lead to adverse effects.^17–22^ The dosages and dose regimens were based on our internal experience and use in monkeys, published literature, and/or consultation with clinicians. In addition, the selected doses were intended to represent feasible anti-inflammatory and immunosuppressant doses, which would not result in adverse toxicity but would result in pharmacodynamic responses relevant to assessing whether immune suppression has any effect on DRG toxicity.^23–29^

After initiation of the 12-month intrathecal study, a 6-month, single-dose, intravenous toxicity study with a 6-week interim cohort was conducted in parallel to the intrathecal NHP studies to evaluate the safety (including DRG toxicity) of intravenous onasemnogene abeparvovec with or without coadministration of prednisolone in juvenile NHPs following additional feedback from the US FDA.

The present publication focuses on microscopic findings in the sensory ganglia (DRG and/or trigeminal ganglion [TG]) following intrathecal onasemnogene abeparvovec administration in NHPs and related biomarkers and study investigations, including molecular localization and electrophysiology (nerve conduction velocity) assessments. Information regarding intravenous dosing in NHPs, including limited microscopic sensory ganglia findings, is also provided. Immunogenicity (anti-AAV9 antibody) and biodistribution assessments by ddPCR were included in the studies but are outside the scope of this publication and will be addressed in separate publications. However, limited data related to these assessments are included to aid in the interpretation of certain immunogenicity and biodistribution data.

## METHODS

### Animals

Cynomolgus monkeys (*Macaca fascicularis*) were acclimated to the test facility for up to 10 weeks before dosing. For the 12-month intrathecal and 6-month intravenous studies, animals were 13–19 months old (males, 1.3–2.5 kg and females, 1.2–1.9 kg). NHPs were 25–34 months old (males, 2.3–4.0 kg and females, 2.6–3.9 kg) for the 13-week mechanistic study.

All procedures were compliant with applicable animal welfare acts and were approved by the local Institutional Animal Care and Use Committee (see Supplementary Appendix for details on NHP origins). Monkeys were screened for the presence of serum total anti-AAV9 antibodies using an enzyme-linked immunosorbent assay method (data not shown). Monkeys with the lowest anti-AAV9 antibody response were selected for inclusion in the study. Because of the endemic presence of anti-AAV9 antibodies in cynomolgus monkeys and the current shortage of monkeys for use in biomedical research, selecting only seronegative monkeys was not possible.

To include an appropriate number of monkeys in the study, the monkeys with highest anti-AAV9 antibody response(s) at screening were assigned to the control group. All groups of animals that were administered onasemnogene abeparvovec had a similar range of anti-AAV9 antibody responses as measured by an electrochemiluminescence immunoassay in samples collected immediately before dose administration (data not shown).

### Study design

In the 12-month study, three groups of NHPs (*n* = 5/sex/group; *n* = 3/sex/group and *n* = 2/sex/group for interim and terminal euthanasia, respectively) of Asian origin were administered a single dose of 1.2 × 10^13^, 3.0 × 10^13^, or 6.0 × 10^13^ vg/animal onasemnogene abeparvovec (human equivalent doses of 1.2 × 10^14^, 3.0 × 10^14^, or 6.0 × 10^14^ vg/patient, respectively, based on a 10-fold cerebrospinal fluid [CSF] scaling factor, as was used for nusinersen,^30^ via slow push intrathecal [lumbar] bolus injection) on day 1 of the dosing phase.

A control group of NHPs (*n* = 5/sex) was administered vehicle control article. The test or control article was administered at a dose volume of 0.80 mL, preceded by 0.20 mL Omnipaque™ 180 contrast agent. The study included an interim (6 weeks postdose) and a terminal evaluation (following 52 weeks of observation postdose; terminal necropsy was conducted on study day 365 or day 1 of week 53).

In the 13-week, non-GLP, mechanistic study, three groups (*n* = 5/sex/group; *n* = 3/sex/group; and *n* = 2/sex/group for interim and terminal euthanasia, respectively) of NHPs of Mauritius origin were administered a single dose of 3 × 10^13^ vg/animal onasemnogene abeparvovec via slow push intrathecal (lumbar) bolus injection on day 1 (see Supplementary Table S1 for study design details). One group (*n* = 5/sex) was administered vehicle control article by intrathecal lumbar puncture on day 1. The test or control article was administered at a dose volume of 0.80 mL, preceded by 0.20 mL Omnipaque 180 contrast agent. Two different anti-inflammatory or immunosuppressive regimens were evaluated: oral prednisolone or a combination of intravenous rituximab and oral everolimus (Table 1).^17–22^

**Table 1.** Dosages and dose regimen of anti-inflammatory and immunosuppressive agents used in the 13-week mechanistic study

Group	Name	Concentration	Dose	Dose Volume	Route	Dosing Days
3	Prednisolone (10 mg as tablets)^a^	1 mg/mL	1 mg/kg/day	1 mL/kg	Oral (gavage)	Day −1 to 29, and then day 31, 33, 35, 37, 39, and 41
4	Rituximab^b^	10 mg/mL	20 mg/kg	2 mL/kg	Intravenous	Every 2 weeks (day −14, day −1, 14, 28, 42, 56, 70, and 84)
4	Everolimus (2.5 mg as tablets)^a^	0.5 mg/mL	0.5 mg/kg/day	1 mL/kg	Oral (gavage)	Daily, week −2 until end of the dosing phase (day 14)

^a^ As these are tablets, they were dissolved in normal drinking water and administered orally by gavage after a visual check for correct dissolution.

^b^ Diphenhydramine is an antihistamine, which has been administered intramuscularly as a premedication 30–60 min before rituximab administration at a concentration of 50 mg/mL, a dose of 4 mg/kg, and a volume of 0.08 mL/kg.

In both intrathecal studies, a contrast agent (Omnipaque 180) was administered separately and immediately before the test article (onasemnogene abeparvovec) to be consistent with the clinical trial procedure to confirm proper placement in the intrathecal space (spinal cord).

In the GLP-compliant 6-month intravenous study in NHPs of Asian origin (*n* = 6/sex/group; *n* = 3/sex/group; and *n* = 3/sex/group for interim and terminal euthanasia, respectively), DRG was evaluated microscopically as part of a complete nonclinical toxicity study after 6 weeks or 6 months of observation following a single dose of onasemnogene abeparvovec (1.1 × 10^14^ vg/kg). A single anti-inflammatory regime of prednisolone at a dosage concentration of 1 mg/kg/day via oral administration once daily starting on day −1 and continuing through day 29, and then on days 31, 33, 35, 37, 39, and 41 of the observation phase was examined (see Supplementary Appendix for details of methods and results).

### Intrathecal onasemnogene abeparvovec administration

Animals were anesthetized at the time of dosing with ketamine and/or medetomidine. Atipamezole was administered as an antidote ∼15 min after dosing. NHPs were maintained in a Trendelenburg tilt (dorsal recumbency with hind legs elevated) for at least 10 min following intrathecal dosing.

### Sample collection

Blood (plasma or serum) and CSF were collected for clinical pathology and analysis of neuronal injury biomarker (neurofilament light chain [NfL]). Tissue samples were collected (Supplementary Table S2), and extensive sampling of the nervous system included collection and processing for light microscopic examination of at least five DRG from each of the cervical, thoracic, and lumbar spinal cord regions and at least two from the sacral region, as well as extensive sampling of the brain, spinal cord, and selected peripheral nerves.

Peripheral nerve sampling differed slightly between the three studies. Specifically, peripheral nerves from the hindlimb (fibular, tibial, sural, and sciatic) were sampled and evaluated in the 12-month intrathecal study. Those same hindlimb nerves, as well as radial and ulnar nerves from the forelimb, were collected and evaluated in the 13-week non-GLP mechanistic intrathecal study.

Finally, in the GLP-compliant 6-month intravenous study, hindlimb (fibular, sciatic, sural, tibial, and medial plantar) and forelimb (radial, ulnar, and median) peripheral nerves were collected and evaluated.

### Nonclinical toxicity assessment

Nonclinical assessments included clinical observations, body weight and qualitative food consumption assessments, ophthalmologic examinations, electrocardiography, and neurologic examinations. Respiration rate assessments, recording of body temperatures, body condition scoring, biodistribution, clinical pathology, and complete macroscopic and microscopic evaluations were obtained. Nonclinical assessments also included, depending on the study design, soluble biomarker for neuronal injury, electrophysiology (neurography or nerve conduction velocity) evaluation, terminal organ weight assessment, molecular localization by *in situ* hybridization (ISH), immunohistochemistry, and biodistribution.

Typically, in a nonclinical study, reversibility is assessed based on the absence or decreased incidence, and/or severity of findings in tissues considered to have a regenerative capacity (*e.g.,* liver or the Schwann cell component of peripheral nerves). For this study, the term “resolution” was applied in the CNS and sensory ganglia to indicate a lack of progression evidenced by the general absence of, and/or decreased incidence and/or severity of, degenerative, necrotic, or inflammatory findings in the neuron components of these tissues, which generally lack a regenerative or neuronogenic response to injury or cell loss in mammals such as NHPs. The presence of aggregates of satellite glial cells represented normal tissue remodeling/glial cell response (scar) subsequent to the loss of degenerate neurons.

### Histology

Paraffin-embedded tissues were routinely processed, sectioned, and stained with hematoxylin and eosin. Organ weight and macroscopic evaluation, microscopic evaluation, interpretation, and pathology peer review were conducted by American College of Veterinary Pathologists board-certified veterinary pathologists experienced in toxicologic pathology. Histopathology grading severity was applied according to test facility standard operating procedures and in accordance with the Society of Toxicologic Pathology Scientific and Regulatory Policy Committee Points to Consider publication by Schafer *et al.*^31^

In brief, a grading scale of 1–5, corresponding to descriptive severities of minimal, slight, moderate, marked, or severe, was applied using International Harmonization of Nomenclature and Diagnostic–recommended terminology in a standard toxicology data collection system (Pristima Version 7.4.2 by Xybion, Princeton, NJ).

For DRG microscopic findings, in which multiple specimens from each geographic region of the vertebral column (cervical, thoracic, lumbar, or sacral) were evaluated, the greatest severity of the finding for that region was recorded, rather than a composite score representing the total area evaluated or the number of normal ganglia versus those with microscopic findings. This decision reflected study objectives to characterize the toxicity of the onasemnogene abeparvovec test article.

### Molecular localization

Molecular localization analysis to detect onasemnogene abeparvovec antisense and sense sequences was undertaken using ISH and paraffin-embedded tissue sections (∼5-μm thick) from selected animals and blocks of spinal cord and DRG at 6 weeks postdose (see Supplementary Appendix for details on methods).

### Neuroelectrophysiologic evaluations

Peripheral nerve function was assessed by neurography (nerve conduction velocity) at 2- and 13-weeks postdose in the 13-week mechanistic study (see Supplementary Appendix for details on methods).

### Prestudy serum anti-AAV9 antibody titers

Prestudy serum anti-AAV9 antibody titers in the three studies are provided in Supplementary Tables S3–S5 (see Supplementary Appendix for details on Methods). The full data set (pretreatment and posttreatment), along with the methodologies used, will be published in a separate follow-up article.

### Onasemnogene abeparvovec DNA concentrations in DRG

The concentrations of onasemnogene abeparvovec DNA in DRG samples in the three studies are provided in Supplementary Tables S6–S8 (see Supplementary Appendix for details on methods). The relationship between preexisting anti-AAV9 antibody titer and distribution of onasemnogene abeparvovec is unknown. The full data set (all tissues and both onasemnogene abeparvovec DNA and mRNA), along with the methodologies used, will be published in a separate, follow-up article.

### Statistical analysis

Data are presented as means and standard deviations. No statistical analysis was performed because of the small number of animals per sex and per group.

### Clinical data evaluation

Data from all onasemnogene abeparvovec clinical trials, irrespective of route of administration (intravenous or intrathecal), were evaluated through May 2021 using the ARGUS safety database. This database contained all postmarketing safety reports received through commercial, managed access program, and early access use. Clinical trial data were also evaluated through May 2021.

## RESULTS

### Safety and tolerability

Intrathecal onasemnogene abeparvovec was clinically well tolerated at all doses tested and was not associated with any clinical findings, including neurobehavioral changes, up to 52 weeks following dose administration of up to 6.0 × 10^13^ vg/animal. No onasemnogene abeparvovec-related changes in qualitative food consumption or body weight, ophthalmic observations, electrocardiography parameters, neurologic findings, respiration rates, body temperatures, cardiac troponin I, organ weights, and/or macroscopic findings were observed. All animals survived to scheduled euthanasia. Similar results were obtained following intravenous administration of onasemnogene abeparvovec in NHPs.

In the 12-month intrathecal NHP study, onasemnogene abeparvovec-related hematology and coagulation changes included minimally to mildly increased absolute monocyte and large unstained cell counts and fibrinogen concentration on days 1 and/or 14 for animals administered ≥3.0 × 10^13^ vg/animal. These changes were transient and not observed at later time points. No onasemnogene abeparvovec-related CSF changes were observed.

In the 13-week non-GLP, mechanistic study, onasemnogene abeparvovec-related hematology (including lymphocyte immunophenotyping), coagulation, and CSF changes included minimally to mildly increased fibrinogen concentrations on days 10, 11, or 12, and/or increased CSF total protein and microalbumin (one female from each group) on day 92 postdose at 3.0 × 10^13^ vg/animal with or without coadministration of prednisolone and/or rituximab plus everolimus (Supplementary Table S9). Fibrinogen concentration changes were not observed at later time points.

Prednisolone-related changes included minimal to mild decrease in total lymphocytes and B and T cells on day 1, and rituximab plus everolimus-related changes included mild decrease in total lymphocytes on days 1, and 10, 11, or 12, mild decreases in T cells on day 1, and marked (100% less than pretest) decrease in B cells throughout observation period indicative of immunosuppression.

In the 6-month intravenous study, onasemnogene abeparvovec-related hematology changes included transient minimally to mildly increased absolute monocyte and large unstained cell counts and mildly to moderately lower platelet counts on days 3–8 at 3.0 × 10^13^ vg/animal with and without coadministration of prednisolone. No onasemnogene abeparvovec-related coagulation or CSF changes were observed. Changes in monocyte counts, large unstained cell counts, and platelets were transient and not observed at later time points.

Any clinical chemistry changes from these studies were outside the scope of this article and will be reported in a different publication.

### Neuronal/axonal injury (NfL) biomarker

**Figure 1.**
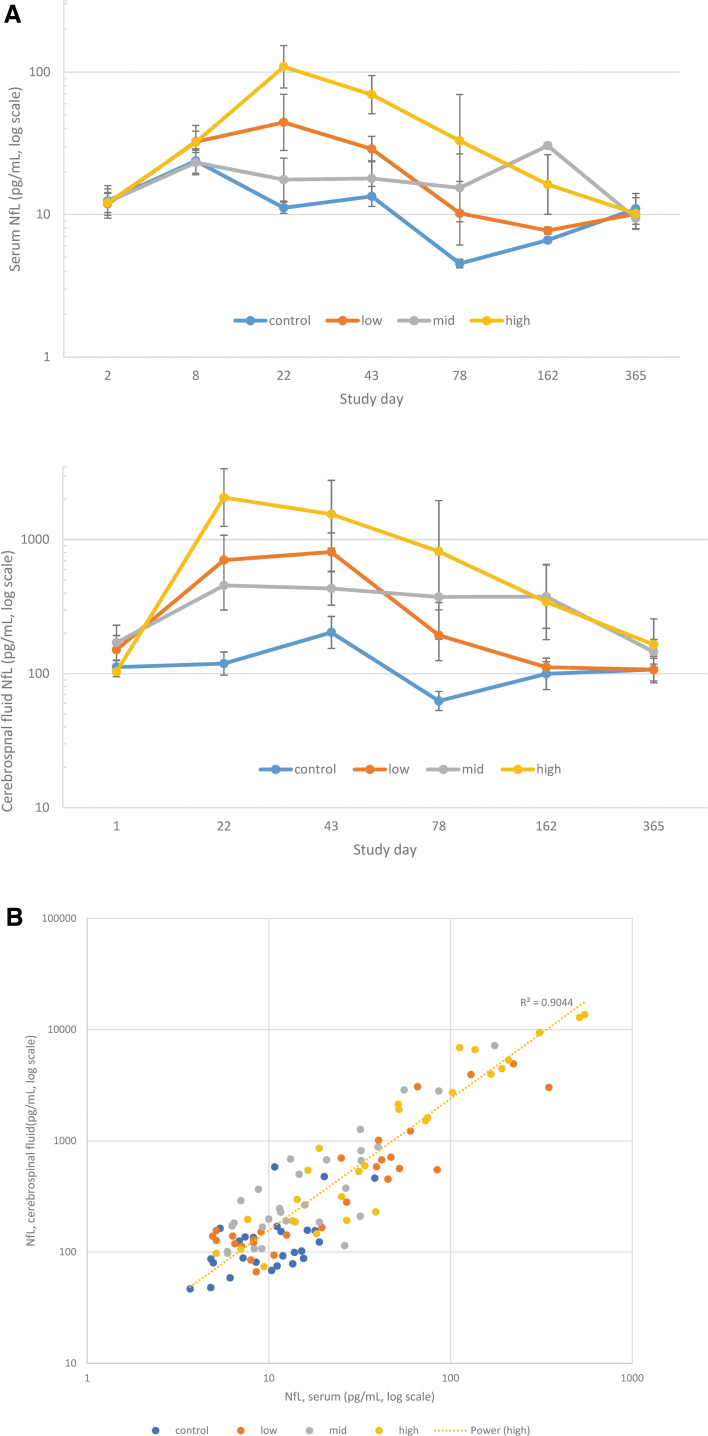
NfL evaluations. **(A)** Serum and CSF NfL concentrations following single-dose intrathecal administration of onasemnogene abeparvovec at low (1.2 × 10^13^ vg/animal), mid (3.0 × 10^13^ vg/animal), or high (6.0 × 10^13^ vg/animal) doses *n* = 5 from pretest to day 43 and *n* = 2 from day 78 onward. **(B)** Correlation of individual serum and CSF NfL concentrations (pg/mL) in male and female NHPs administered intrathecal onasemnogene abeparvovec. CSF, cerebrospinal fluid; NfL, neurofilament light chain; NHPs, nonhuman primates.

In the 12-month intrathecal NHP study, onasemnogene abeparvovec-related trends toward increased serum and CSF NfL, a marker of axonal injury, were observed in individual animals from all dose groups on day 22, with trends toward predose and vehicle control concentrations by day 163 and resolution by day 365 (Fig. 1A). A correlation between CSF and serum NfL concentrations was observed (*R*^2^ = 0.9044; Fig. 1B).

In the 13-week intrathecal NHP study, individual animal increases in plasma and CSF NfL concentrations were observed following administration of onasemnogene abeparvovec alone or with immunosuppressants (prednisolone or rituximab/everolimus) at different intervals between days 12 and 92 postdose (Fig. S1). Similar NfL trends were observed in the 6-month intravenous studies (Supplementary Fig. S2).

### Microscopic DRG and TG findings

#### Thirteen-week intrathecal mechanistic study

In the 2-week interim cohort, onasemnogene abeparvovec-related nervous system microscopic findings were limited to the DRG and included minimal to marked mononuclear cell inflammation, minimal to moderate neuronal degeneration, and minimally to slightly increased satellite glial cells. Mononuclear cell inflammation was characterized by variable and increased numbers of mixed mononuclear cells associated with areas of tissue and/or neuronal degeneration and/or the presence of cellular debris. Neuronal degeneration was characterized by small and/or irregularly shrunken neuronal cell bodies with scalloped borders and an indistinct or absent nucleus with or without cellular debris, typically surrounded by mononuclear cells.

Secondary findings included minimal to slight axon degeneration and minimal mononuclear cell infiltrates in the adjacent spinal root and/or spinal nerve (Table 2 and Supplementary Table S10).

**Table 2.** Effect of immunosuppression on onasemnogene abeparvovec-related microscopic dorsal root ganglion findings at 2 and 13 weeks of observation postintrathecal dosing for females in the 13-week mechanistic study

Tissue/finding	Females							
Dose (vg/animal)	0	0	3 × 10^13^	3 × 10^13^	3 × 10^13^	3 × 10^13^	3 × 10^13^	3 × 10^13^
Prednisolone (mg/kg)	0	0	0	0	1	1	0	0
Rituximab (mg/kg)	0	0	0	0	0	0	20	20
Everolimus (mg/kg)	0	0	0	0	0	0	0.5	0.5
Necropsy/study day	15	92	15	92	15	92	15	92
**Ganglion, cervical dorsal root**								
Number examined	3	2	3	2	3	2	3	2
Inflammation, mononuclear cell								
Total number affected	0	0	1	1	0	1	1	1
Minimal	0	0	0	0	0	1	1	0
Slight	0	0	1	0	0	0	0	1
Moderate	0	0	0	1	0	0	0	0
Degeneration, neuron								
Total number affected	0	0	2	1	0	2	1	1
Minimal	0	0	1	0	0	2	1	0
Slight	0	0	1	1	0	0	0	1
Degeneration, axon, spinal root/spinal nerve								
Total number affected	0	0	0	1	0	0	0	0
Slight	0	0	0	1	0	0	0	0
Satellite glial cells, increased								
Total number affected	0	N/A	2	N/A	2	N/A	1	N/A
Minimal	0	N/A	2	N/A	2	N/A	1	N/A
Satellite glial cell, increased/neuronal cell loss								
Total number affected	N/A	0	N/A	1	N/A	0	N/A	0
Minimal	N/A	0	N/A	1	N/A	0	N/A	0
Slight	N/A	0	N/A	0	N/A	0	N/A	0
**Spinal cord, cervical**								
Number examined	3	2	3	2	3	2	3	2
Degeneration, axon, dorsal funiculus								
Total number affected	0	0	0	2	0	2	1	2
Minimal	0	0	0	0	0	2	1	1
Slight	0	0	0	1	0	0	0	—
Moderate	0	0	0	1	0	0	0	1
Gliosis, dorsal funiculus, white matter								
Total number affected	0	0	0	0	0	1	0	0
Minimal	0	0	0	0	0	1	0	0
Gliosis, gray matter								
Total number affected	0	0	0	1	0	0	0	0
Minimal	0	0	0	1	0	0	0	0
Degeneration, neuron								
Total number affected	0	0	0	1	0	0	0	0
Minimal	0	0	0	1	0	0	0	0

N/A, not available.

**Figure 2.**
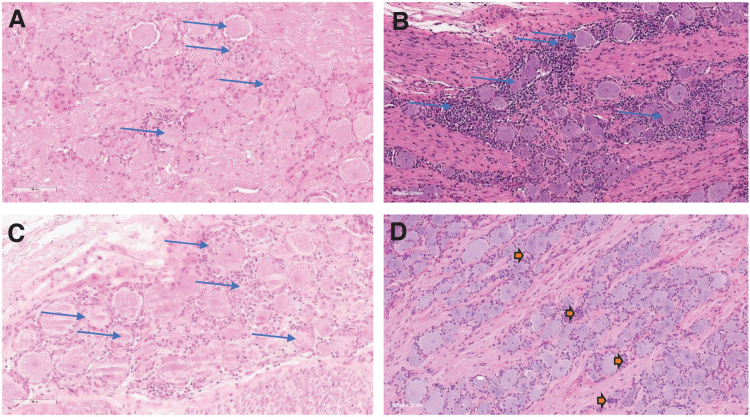
Microscopic DRG findings at 2, 6, 13, and 52 weeks of observation postdose. H&E-stained, formalin-fixed, paraffin-embedded cervical dorsal root (spinal) ganglia from NHPs dosed 3 × 10^13^ vg/animal by intrathecal lumbar puncture in a dose volume of 0.80 mL. **(A)** Slight neuron degeneration and mononuclear cell inflammation with minimal increased satellite glial cells at 2 weeks postdose. **(B)** Moderate neuron degeneration and mononuclear cell inflammation at 6 weeks postdose. **(C)** Slight neuron degeneration and moderate mononuclear cell inflammation at 13 weeks postdose. **(D)** Aggregates of satellite glial cells in the cell body rich area of the ganglia at 52 weeks postdose, representing areas of scar or tissue remodeling following neuronal loss because of degeneration. Magnification, 200 × . DRG, dorsal root ganglion; H&E, hematoxylin and eosin.

**Figure 3.**
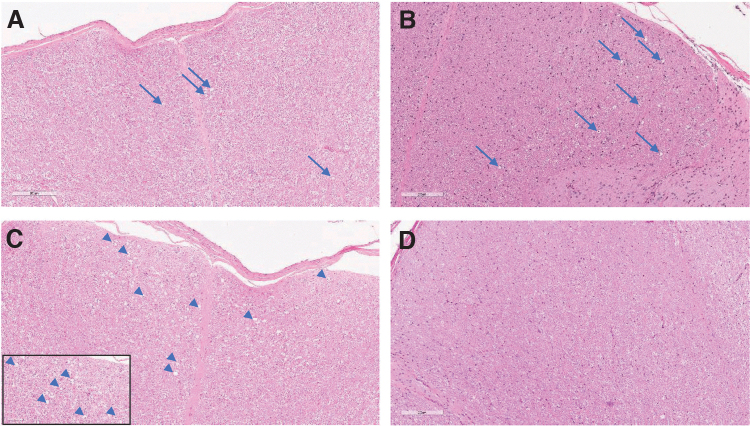
Microscopic spinal cord findings at 2, 6, 13, and 52 weeks of observation postdose. H&E-stained, formalin-fixed, paraffin-embedded cervical spinal cord from NHPs dosed 3 × 10^13^ vg/animal by intrathecal lumbar puncture in a dose volume of 0.80 mL. **(A)** Cervical spinal cord demonstrating minimal axon degeneration of the dorsal funiculus at 2 weeks postdose. **(B)** Slight axon degeneration at 6 weeks postdose. **(C)** Cervical spinal cord demonstrating moderate axon degeneration of the dorsal funiculus at 13 weeks postdose (*inset* magnification, 400 × ). **(D)** Absence of microscopic findings in the dorsal funiculus at 52 weeks postdose. Magnification, 200 × .

Axon degeneration was characterized variably by one or more of the following: dilated spaces (formerly occupied by myelin sheaths and axons), swollen axons, cellular debris, and/or macrophages. Mononuclear cell infiltrates were characterized by increased number of mononuclear cells that were not associated with tissue degeneration or damage compared with mononuclear cell inflammation. In general, these findings were reported as similar in incidence and severity in animals administered 3 × 10^13^ vg/animal onasemnogene abeparvovec alone or with prednisolone or rituximab/everolimus. At 13 weeks postdose, onasemnogene abeparvovec-related microscopic findings were observed in the DRG and/or TG (general proprioception and somatic afferent sensory ganglia) (Figs. 2 and 3, Table 2, and Supplementary Table S10).

Microscopic findings in these tissues were the same as observed at 2 weeks, and included neuronal cell loss, minimal to slight satellite glial cell aggregates, minimal to moderate mononuclear cell infiltrates, and/or slight axonal degeneration. The incidence and severity of the DRG and/or TG microscopic findings at 2- and 13-weeks postdose were comparable.

#### Twelve-month intrathecal GLP study

Similar to the interim and terminal necropsy findings in the 13-week intrathecal mechanistic study, microscopic onasemnogene abeparvovec-related findings were observed in the DRG and/or TG following at 6 weeks postintrathecal dosing and generally consisted of minimal to moderate mononuclear cell inflammation, minimal to moderate neuronal degeneration, minimal to slight mononuclear cell infiltrates, and/or minimal hemorrhage for animals administered ≥1.2 × 10^13^ vg/animal (Fig. 2, Table 3, and Supplementary Table S11).

**Table 3.** Summary incidence and severity onasemnogene abeparvovec-related microscopic findings in the dorsal root ganglia and trigeminal ganglia at 6 and 52 weeks of observation postintrathecal dosing for females in the 12-month GLP-compliant study

Tissue/finding	Females							
Dose (vg/animal in 0.80 mL volume)	0	0	1.2 × 10^13^	1.2 × 10^13^	3 × 10^13^	3 × 10^13^	6 × 10^13^	6 × 10^13^
Number examined	3	2	3	2	3	2	3	2
Necropsy/study day	43	365	43	365	43	365	43	365
**Ganglion, cervical dorsal root**								
Inflammation, mononuclear cell								
Total number affected	0	0	2	1	1	0	2	1
Minimal	0	0	2	1	0	0	0	1
Slight	0	0	0	0	1	0	2	0
Moderate	0	0	0	0	0	0	0	0
Degeneration, neuron								
Total number affected	0	0	2	1	1	0	2	1
Minimal	0	0	2	1	0	0	1	1
Slight	0	0	0	0	1	0	1	0
Moderate	0	0	0	0	0	0	0	0
Degeneration, axon, spinal root/spinal nerve								
Total number affected	0	0	0	0	1	0	2	0
Minimal	0	0	0	0	0	0	2	0
Slight	0	0	0	0	1	0	0	0
Infiltrate, mononuclear cell, spinal root/spinal nerve								
Total number affected	0	0	0	0	1	0	1	0
Minimal	0	0	0	0	1	0	1	0
Slight	0	0	0	0	0	0	0	0
Aggregate, satellite glial cell								
Total number affected	0	0	0	1	0	0	0	2
Minimal	0	0	0	1	0	0	0	2
**Ganglion, thoracic dorsal root**								
Inflammation, mononuclear cell								
Total number affected	0	0	0	1	0	0	0	1
Minimal	0	0	0	1	0	0	0	1
Degeneration, neuron								
Total number affected	0	0	0	1	0	0	0	1
Minimal	0	0	0	1	0	0	0	1
Aggregate, satellite glial cell								
Total number affected	0	0	0	0	0	1	0	0
Minimal	0	0	0	0	0	1	0	0
**Ganglion, lumbar dorsal root**								
Inflammation, mononuclear cell								
Total number affected	0	0	1	0	0	0	3	0
Minimal	0	0	0	0	0	0	2	0
Slight	0	0	0	0	0	0	0	0
Moderate	0	0	1	0	0	0	1	0
Degeneration, neuron								
Total number affected	0	0	1	0	0	0	2	0
Minimal	0	0	0	0	0	0	1	0
Slight	0	0	1	0	0	0	1	0
Moderate	0	0	0	0	0	0	0	0
Degeneration, axon, spinal root/spinal nerve								
Total number affected	2	0	2	0	1	0	3	0
Minimal	2	0	1	0	0	0	1	0
Slight	0	0	1	0	1	0	2	0
Moderate	0	0	0	0	0	0	0	0
Infiltrate, mononuclear cell, spinal root/spinal nerve								
Total number affected	0	0	2	0	1	0	1	0
Minimal	0	0	1	0	0	0	1	0
Slight	0	0	1	0	1	0	0	0
**Ganglion, sacral dorsal root**								
Inflammation, mononuclear cell								
Total number affected	0	0	2	0	1	1	2	1
Minimal	0	0	1	0	1	1	1	1
Slight	0	0	0	0	0	0	1	0
Moderate	0	0	1	0	0	0	0	0
Degeneration, neuron								
Total number affected	0	0	1	0	1	1	2	1
Minimal	0	0	0	0	1	1	1	1
Slight	0	0	0	0	0	0	1	0
Moderate	0	0	1	0	0	0	0	0
Degeneration, axon, spinal root/spinal nerve								
Total number affected	0	0	1	0	0	0	2	0
Minimal	0	0	0	0	0	0	0	0
Slight	0	0	1	0	0	0	0	0
Moderate	0	0	0	0	0	0	2	0
Infiltrate, mononuclear cell, spinal root/spinal nerve								
Total number affected	0	0	1	0	0	0	2	0
Minimal	0	0	1	0	0	0	2	0
**Ganglion, trigeminal**								
Inflammation, mononuclear cell								
Total number affected	0	0	1	0	1	1	2	0
Minimal	0	0	0	0	1	1	2	0
Slight	0	0	1	0	0	0	0	0
Degeneration, neuron								
Total number affected	0	0	1	0	1	1	2	0
Minimal	0	0	1	0	1	1	2	0

After 52 weeks of observation postintrathecal dosing, microscopic DRG and/or TG findings were limited to minimal neuronal degeneration, mononuclear cell inflammation, and/or satellite glial cell aggregates in animals administered ≥1.2 × 10^13^ vg/animal (in the TG/nerve, findings were limited to animals administered ≥3 × 10^13^ vg/animal).

Satellite glial cell aggregates (historically sometimes referred to as residual nodules [nodules of Nageotte]) were characterized by small clusters of closely packed, disorganized glial cells in the ganglia and were consistent with normal tissue remodeling in the nervous system and reflective of a glial response (scar) related to resolution in this tissue following neuronal degeneration that resulted in loss of the affected neuron. The incidence and/or severity of these microscopic findings was generally lower compared with animals examined at 6 weeks postdose (Fig. 2, Table 3, and Supplementary Table S11). These changes were not considered progressive when evaluated after 52 weeks of observation or associated with any in-life neurologic findings (see Supplementary Appendix for details).

#### Six-month intravenous GLP study

Minimal to slight neuronal degeneration and mononuclear cell inflammation were the only microscopic DRG and/or TG findings observed following intravenous onasemnogene abeparvovec dosing (1.1 × 10^14^ vg/kg) in NHPs at 6 weeks and 6 months postdose (Supplementary Tables S12 and S13).

### Microscopic findings in the spinal cord and peripheral nerves

#### Thirteen-week intrathecal mechanistic study

Onasemnogene abeparvovec-related microscopic findings were not observed in the spinal cord or peripheral nerves at 2 weeks postintrathecal dosing (3 × 10^13^ vg/animal). At 13 weeks postdose, onasemnogene abeparvovec-related microscopic findings in the spinal cord and dorsal spinal nerve root/spinal nerves that were considered secondary to the DRG findings observed at the 2 week postdose necropsy consisted of minimal to moderate axonal degeneration in the dorsal (posterior) funiculus of the spinal cord and/or the dorsal spinal cord nerve roots, minimal gliosis in the dorsal funiculus, and minimal to slight mononuclear cell infiltrates in the dorsal spinal nerve roots (Fig. 3, Table 2, and Supplementary Table S10).

The dorsal spinal nerve roots are composed of axons originating from the sensory neurons of the DRG, which then bifurcate and centrally form the ascending sensory tracts of the dorsal funiculus or dorsal/posterior white matter tract and peripherally form the sensory nerves. Microscopic findings of inflammation and/or degeneration in the DRG are expected to subsequently manifest in these nerve root and spinal cord regions, as well as peripheral nerves.

Compared with findings considered secondary to the sensory ganglia, minimal neuronal degeneration and/or minimal to slight gliosis were observed in the ventral gray matter (anterior gray column; motor neurons) of the cervical, thoracic, and/or lumbar spinal cord of one male and two females administered onasemnogene abeparvovec alone or with prednisolone. Slight or moderate severity neuronal degeneration and gliosis in the spinal cord were sampled at the location of the intrathecal injection site.

These findings were considered separate to the DRG findings, but may also be related to greater transduction of some cells in this tissue. Microscopic findings in the ventral motor neurons were not associated with any in-life or neurologic observations and may be of similar pathogenesis to the DRG findings in that they are subsequent to greater transduction of the AAV construct and/or greater expression of the transgene product. However, whether the response is specific to the transgene protein product is not clear.

At 13 weeks postdose, onasemnogene abeparvovec-related microscopic findings in peripheral nerves consisted of minimal to marked axonal degeneration and minimal mononuclear cell infiltrates. Peripheral nerve axon degeneration was generally considered secondary to DRG neuronal degeneration.

#### Twelve-month intrathecal GLP study

After 6 weeks, microscopic findings in the spinal cord consisted of minimal to moderate axon degeneration and minimal gliosis in the dorsal funiculus, minimal or slight axon degeneration in the dorsal spinal nerve roots, and minimal to slight mononuclear cell infiltrate in the spinal nerve/spinal nerve roots. In addition, minimal to moderate neuronal degeneration and/or minimal to slight gliosis was observed in the ventral gray matter (anterior gray column) of the spinal cord in a few animals administered ≥3.0 × 10^13^ vg/animal (Fig. 2, Table 3, and Supplementary Table S14).

Although this finding was uncommon and was distributed more often in the lumbar or sacral spinal cord (potentially in the area of the intrathecal administration site), the distribution of degenerate neurons in the ventral gray column, but not the dorsal gray column, in multiple sections of the spinal cord in one of two 6 × 10^13^ vg/animal males at 6 weeks postdose rendered injury because of intrathecal injection unlikely. Therefore, these findings were considered onasemnogene abeparvovec related. No correlative in-life clinical or neurologic observations were reported in these animals.

After 52 weeks of observation, minimal to slight axonal degeneration and/or minimal gliosis in the dorsal funiculus was present for animals administered ≥3.0 × 10^13^ vg/animal (Fig. 3 and Supplementary Table S15). Minimal gliosis in the ventral and dorsal gray matter of the thoracic and lumbar spinal cord of one female administered 6 × 10^13^ vg/animal was also observed. These microscopic findings, which were not associated with any in-life clinical or neurologic observations, were noted at lower incidences and/or severities compared with those at 6 weeks postdose for animals administered ≥3.0 × 10^13^ vg/animal and were comparable between concurrent controls and animals administered 1.2 × 10^13^ vg/animal.

Onasemnogene abeparvovec-related microscopic findings in the peripheral nerves consisted of minimal to slight axon degeneration in animals administered ≥1.2 × 10^13^ vg/animal at 6 weeks postdose with no dose relationship (Supplementary Table S16). These microscopic findings in peripheral nerves were not observed following 52 weeks of observation postdose.

#### Six-month intravenous GLP study

Onasemnogene abeparvovec-related microscopic findings in the spinal cord following intravenous administration consisted generally of minimal severity axon degeneration, with one male observed with slight severity axon degeneration, in the ventral and lateral funiculi (with relative sparing of the dorsal funiculus) and minimal gliosis in the spinal cord white matter at 6 weeks postdose.

Minimal axon degeneration was observed at a similar incidence and severity in concurrent female controls and onasemnogene abeparvovec-dosed females at the interim necropsy, with no onasemnogene abeparvovec-related increase in incidence or severity in females at this 6-week time point. At the end of the 6-month observation period, axon degeneration in the spinal cord was still present at similar (males) or lower (females) incidence and severity compared with the end of the 6-week observation period, supporting a lack of progression of the findings.

No clinical observations, neurologic examination findings, or other correlations were observed for these minimal or slight axon findings in the ventral or lateral funiculi.

No onasemnogene abeparvovec-related microscopic finding was noted in peripheral nerves (fibular, sciatic, sural, tibial, medial plantar, radial, ulnar, and median) following intravenous administration of onasemnogene abeparvovec at either 6-weeks or 6-months postdose.

### ISH for onasemnogene abeparvovec vector sequences

ISH for onasemnogene abeparvovec antisense and sense gene probes was conducted on selected sections of spinal cord and selected DRG from two males administered intrathecally 6.0 × 10^13^ vg/animal and two concurrent controls (females) to localize vector nucleic acid. Analysis included appropriate positive and tissue quality control (*macaca mulatta peptidyl-prolyl cis-trans isomerase B*) and negative control (*dihydrodipicolinate reductase*) genes.

**Figure 4.**
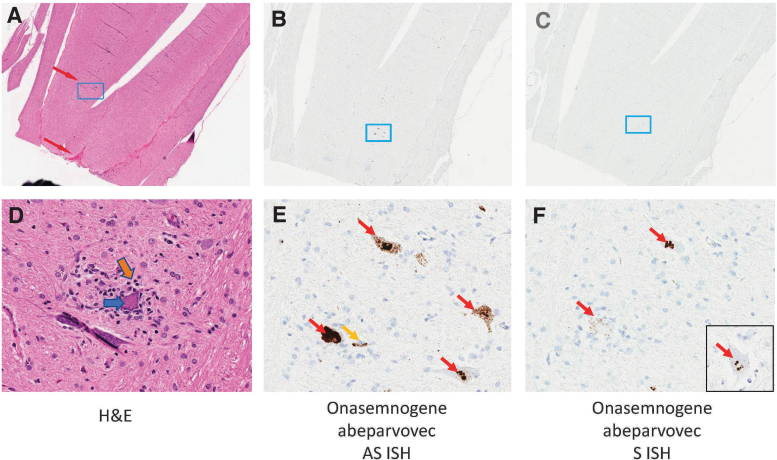
Molecular localization of onasemnogene abeparvovec. Vector expression was detected in lower motor neurons of spinal cord following intrathecal administration of onasemnogene abeparvovec (6.0 × 10^13^ vg/animal) at 6 weeks postdose. **(A, D)** H&E-stained tissues. **(A)** *Top red arrow* indicates the ventral (anterior) *gray* horn and *lower red arrow* indicates the ventral median fissure with ventral spinal artery and vein. ISH using an **(B, E)** onasemnogene abeparvovec antisense probe or **(C, F)** onasemnogene abeparvovec sense probe. Cytoplasmic sense probe signal within individual lower motor neuron **(***inset* **F)**. Magnification: *top panels*, 2 × and *bottom panels*, 32 × . ISH, *in situ* hybridization.

ISH for onasemnogene abeparvovec using the antisense probe on sections of spinal cord from the two males with microscopic neuronal degeneration/satellitosis in the ventral gray matter noted at 6 weeks postdose detected moderate to high signal concentrations consistent with high concentrations of vector transcript in a subset of scattered individual neurons with morphologic features of degeneration and satellitosis compared with adjacent unaffected neurons (Fig. 4).

ISH with onasemnogene abeparvovec sense probes conducted on sequential sections of spinal cord detected lesser concentrations of cytoplasmic signal consistent with the presence of double-stranded ribonucleic acid. In regions of the spinal cord without microscopic findings, lesser concentrations of antisense and sense signals were observed and characterized by small, punctate nuclear and cytoplasmic signals. Sections from concurrent control animals did not detect signal from antisense or sense onasemnogene abeparvovec probes. Positive and negative control probes returned expected results.

In DRG from the two males with microscopic neuronal degeneration at 6 weeks postdose, ISH for onasemnogene abeparvovec using the antisense probe detected moderate to high signal concentrations consistent with high concentrations of vector transcript in individual neurons with morphologic features of degeneration compared with adjacent unaffected neurons (Supplementary Fig. S3A). ISH for onasemnogene abeparvovec using the sense probe detected moderate signal in the cytoplasm of individual neurons with similar morphologic features of degeneration, but the signal was much sparser than the antisense signal (Supplementary Fig. S3B).

### Peripheral nerve function following intrathecal onasemnogene abeparvovec

**Figure 5.**
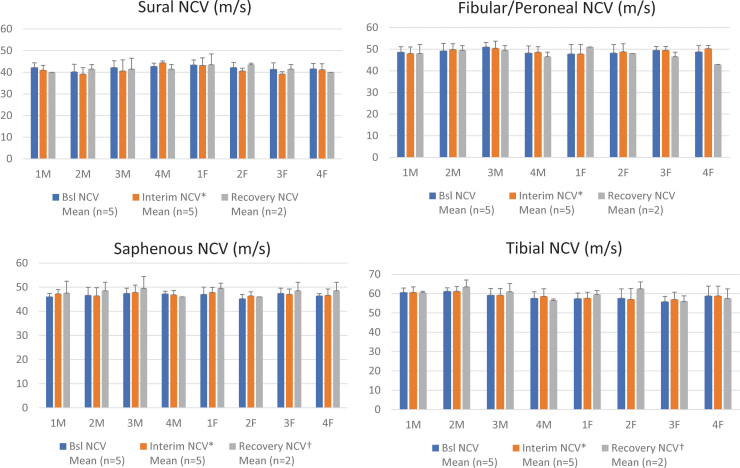
Neuroelectrophysiologic evaluations. **(A)** Sural (sensory) nerve conduction, **(B)** fibular/peroneal (sensory) nerve conduction, **(C)** saphenous (sensory) nerve conduction, and **(D)** tibial (motor) nerve conduction in males (M) and females (F) was determined at baseline, 2 weeks postdose (interim) and 13 weeks postdose (recovery) for NHPs in all four groups treated with onasemnogene abeparvovec. Group 1 was administered vehicle control item and contrast agent only. Group 2 was administered onasemnogene abeparvovec (3 × 10^13^ vg/animal) without immunosuppression. Group 3 was administered onasemnogene abeparvovec (3 × 10^13^ vg/animal) along with oral prednisolone (1 mg/kg). Group 4 was administered onasemnogene abeparvovec (3 × 10^13^ vg/animal) and a combination of intravenous rituximab and oral everolimus. NCV are all L (m/s). NCV, nerve conduction velocity.

Neuroelectrophysiologic evaluations assessing peripheral nerve function following intrathecal onasemnogene abeparvovec (3 × 10^13^ vg/animal) with and without immunosuppression (prednisolone or rituximab and everolimus) in the 13-week mechanistic study demonstrated that mean peripheral sensory nerve conduction was within normal functional range at 2- and 13-weeks postdose. No physiologically relevant decreases were observed compared with concurrent controls in the sural, peroneal, and saphenous sensory nerves. No velocity decreases in the tibial motor function and ascending motor pathways were observed compared with concurrent controls (Fig. 5). Similar results were obtained following intravenous onasemnogene abeparvovec administration (1.1 × 10^14^ vg/kg) in NHPs (see Supplementary Appendix for details of results).

### Onasemnogene abeparvovec biodistribution and immunogenicity following intrathecal administration

After intrathecal administration, onasemnogene abeparvovec was distributed widely to all tissues analyzed, with pronounced transduction of the spinal cord and DRG (data not shown). A rapid and robust humoral immune response to the AAV capsid was observed in both serum and CSF (data not shown).

### Clinical safety data

As of May 2021, 102 patients received intravenous onasemnogene abeparvovec and 32 patients received intrathecal onasemnogene abeparvovec in clinical trials. In clinical trials, pediatric neuromuscular experts were trained to conduct neurologic assessments for all children, including infants documented in serial comprehensive examinations, to evaluate for signs or symptoms of ganglionopathy. Also, all reported that adverse events were reviewed to evaluate for signs or symptoms of ganglionopathy.

Although self-reporting may be limited as these patients were infants, parental reports and investigator examinations were obtained to assess for ganglionopathy. No events suggestive of ganglionopathy were reported, and a review of documented neurologic and physical examination data in clinical studies demonstrated no findings consistent with ganglionopathy. Based on a review of safety data from the ARGUS safety database, no events of ganglionopathy have been reported as of May 2021.

## DISCUSSION

For juvenile cynomolgus monkeys, intrathecal onasemnogene abeparvovec administration at doses up to 6.0 × 10^13^ vg/animal was well tolerated and not associated with any in-life clinical or neurologic observations up to 52 weeks postdose. Adverse microscopic findings in the DRG and TG, including neuron degeneration, were considered nonprogressive with a trend toward resolution based on lower incidence and minimal severity of microscopic observations after 52 weeks of observation. In addition, microscopic findings, including aggregates of satellite glial cells, were considered consistent with tissue remodeling over time following loss of the affected (degenerate) neurons.

Neuronal cell degeneration/neuron loss is generally not considered a reversible finding, because neurons are not thought to have regenerative capacity in NHPs (compared with reported neurogenesis in dogs and rodents).^32–36^

Instead, in the 12-month NHP study, “resolution” was used to connote a decrease in the incidence and/or severity of the microscopic neuronal degeneration in the DRG and TG as well as the absence of related/secondary axonal degeneration in the examined peripheral nerves and central axonal projections of this sensory neuron population (dorsal funiculus of the spinal cord).

Satellite glial cell aggregates represented a tissue remodeling/glial cell response (scar) subsequent to the loss of degenerative neurons and were not considered adverse in and of themselves but rather interpreted as evidence of quiescence/nonprogression of the microscopic neuronal and/or inflammatory findings in the sensory ganglia (DRG and TG) after 52 weeks of observation without any in-life neurologic or clinical onasemnogene abeparvovec-related findings.

At the interim necropsy, 6 weeks after the single intravenous dose, microscopic spinal cord findings of minimal, or rarely slight, severity axon degeneration were increased in incidence and severity (slight in one animal) in onasemnogene abeparvovec-dosed males compared with concurrent controls, but were similar in controls and onasemnogene abeparvovec–dosed females. At the terminal necropsy, 6 months after dosing, incidence in females was lower compared with the interim necropsy, while incidence in males was similar. These findings were of uncertain pathogenesis, because minimal axon degeneration may be observed in undosed NHPs.^37^

The onasemnogene abeparvovec-related microscopic DRG/TG findings observed in the NHP studies were comparable with those observed with several other intrathecally or intravenously administered AAV (AAV9, AAV1, and AAV5) gene therapies that used assorted transgenes, confirming that these findings were onasemnogene abeparvovec related.^38–40^ As with the present studies, these microscopic findings were typically without corresponding clinical signs and did not demonstrate a dose response.^39^

Our primary microscopic findings consisted of degeneration of DRG neurons with mononuclear inflammation. Associated axon degeneration and related microscopic findings (gliosis, mononuclear cell infiltrate) were observed in structures composed, at least in part, of the axon branches from the sensory DRG neurons that form the dorsal nerve roots, ascending spinal and bulbar nerve tracts, and sensory axons in peripheral nerves. This degeneration occurred within 2–6 weeks of intrathecal administration.

The clinically silent nature of this microscopic finding was considered related to the limited, multifocal distribution and the relatively low extent and magnitude of the affected sensory ganglia (DRG and TG). Only a small section of the overall general proprioceptive and somatic afferent sensory ganglia was affected, in a system with substantial redundancy, given the evolutionary importance of these key sensory inputs for survival.^41^ These findings were nonprogressive and demonstrated morphologic features of expected tissue remodeling (aggregates of satellite glial cells and/or neuronal loss).

AAV-mediated DRG changes were likely multifactorial and potentially driven by increased sensitivity to greater transduction of individual DRG neurons by some AAV serotypes, most notably AAV9 and close analogs, although a contribution by specific transgene or transgene products may impact some circumstances.^39^

Molecular localization (by ISH) for antisense and sense onasemnogene abeparvovec probes and routine light microscopic findings from NHP studies also support this hypothesis, demonstrating high transgene vector expression in the affected (degenerate) DRG sensory neurons (lumbar and sacral) of two males administered onasemnogene abeparvovec at a dose of 6 × 10^13^ vg/animal (Supplementary Fig. S3). High concentrations of antisense signal were accompanied by lower concentrations of cytoplasmic sense signal. In contrast, low expression was present in adjacent unaffected neurons (lacking any morphologic features of degeneration).

Together, these patterns of high vector transcript demonstrated by ISH collocated with morphologic findings of degeneration, necrosis, satellitosis, and/or mononuclear cell inflammation are consistent with a common pathogenesis driven by high concentrations of vector transduction and transgene expression, resulting in neuronal degeneration in the DRG in animals administered ≥1.2 × 10^13^ vg/animal.

Although several other factors, including vector dose, route of administration, and age at dosing, may play a role in AAV9-mediated microscopic DRG findings in NHPs, activation of the innate immune response in transduced cells was not excluded.^38,39,42,43^ In fact, the sense probes for onasemnogene abeparvovec performed on sequential sections of nervous tissue by ISH detected cytoplasmic signal consistent with the presence of double-stranded ribonucleic acid, which may elicit an innate immune response through retinoic acid-inducible gene I-like receptor signaling pathways. Sensory neurons in the DRG were surrounded by satellite glial cells, scant numbers of resident tissue macrophages, and T and B lymphocytes,^44–46^ which were associated with AAV-mediated microscopic DRG findings.^38,39^

Systemic immunosuppression, including 100% B cell depletion at all time points, did not prevent, attenuate, and/or mitigate the incidence or severity of onasemnogene abeparvovec-related microscopic DRG findings in NHPs dosed with 3 × 10^13^ vg/animal by intrathecal lumbar injection, suggesting that primary adaptive immune responses may not be a critical mediator in the pathogenesis of microscopic DRG (or TG) findings. The microscopic and molecular localization findings reported here are consistent with a published study, in which immunosuppression (mycophenolate mofetil and rapamycin) administered to NHPs treated with unmodified AAV9 did not alleviate DRG damage.^42^

NfL has been used in the clinic to monitor for neuronal/axonal injury and has recently been considered for monitoring of clinical response to molecular therapies.^47^ Minor to moderate transient increases in serum or CSF NfL concentrations were observed at different time points between days 12 and 92 postdose, decreasing toward baseline, with eventual resolution by day 365 following intrathecal onasemnogene abeparvovec administration in NHPs. There appears to be a correlation between the transient NfL increases and the onset of microscopic DRG findings observed at necropsy in NHPs in nonclinical safety studies. Compared with patients with neurodegenerative conditions, such as SMA, species used in nonclinical safety assessments are outbred, clinically healthy animals.

This enables identification of test article-related findings in the absence of concurrent disease or comorbid conditions, which is not possible in a human population. Serum or CSF NfL findings associated with administration of intrathecal onasemnogene abeparvovec administration in NHPs as reported here should be considered in the context of this nonclinical, nondiseased test system, and appropriate caution should be applied for any potential translation to human patients.

No neuroelectrophysiologic changes (assessed via peripheral nerve function) were reported following intrathecal or intravenous onasemnogene abeparvovec administration in NHPs. Mean peripheral sensory nerve conduction remained within the normal functional range, indicating no correlation between the DRG changes and peripheral nerve function. This finding is consistent with the lack of clinical or neurologic observations during in-life in animals. The generally low percentage of affected neurons in the DRG with onasemnogene abeparvovec-related microscopic findings, and the degree of redundancy and overlap associated with innervation of the dermatomes of the sensory system, may further explain the lack of in-life findings.

The translational relevance of DRG pathology in humans is currently unknown. The medical literature includes one reported case of putative microscopic DRG findings in a 22-year-old male with amyotrophic lateral sclerosis and mutations in the superoxide dismutase 1 (*SOD1*) gene who received a single intrathecal infusion of AAV-miR-SOD1 (4.2 × 10^14^ vg).^48^ However, the published information was limited, and the authors were not able to determine if the findings were because of gene therapy or the underlying disease state. This case along with another unpublished case is presented in more detail in the Supplemental Results. In the present study, evaluation of available clinical safety data after intravenous and intrathecal onasemnogene abeparvovec dosing in clinical trials reported no clinical sensory neuropathy findings as of May 2021.

## CONCLUSIONS

The nonclinical microscopic DRG findings reported in the NHP studies trended toward resolution defined by lower incidence and severity, and some findings were consistent with tissue remodeling (scar) following a 52-week observation period. These observations support the nonprogressive nature of the nervous system findings, which were not associated with clinically detectable neurologic findings or interpretations. In addition, microscopic DRG findings were not mitigated with coadministration of anti-inflammatory or immunosuppressive regimens. The pathogenesis is possibly a consequence of increased vector genome transduction and/or transgene expression.

For translational applicability to patients, analysis of clinical data in the onasemnogene abeparvovec clinical trials, using the intrathecal or intravenous route of administration, as well as review of postmarketing safety data,^15^ did not reveal any clinical findings of ganglionopathy. Therefore, the clinical relevance of the DRG findings in NHP studies associated with intrathecal or intravenous administration of AAV vector gene therapies remains unknown. This potential risk will be monitored through usual clinical safety surveillance.

## Supplementary Material

Supplemental data

Supplemental data

Supplemental data

Supplemental data

Supplemental data

Supplemental data

Supplemental data

Supplemental data

Supplemental data

Supplemental data

Supplemental data

Supplemental data

Supplemental data

Supplemental data

Supplemental data

## DATA-SHARING STATEMENT

Novartis is committed to sharing clinical trial data with external researchers and has been doing so voluntarily since 2014. Novartis was the third member to join ClinicalStudyDataRequest.com (CSDR), which is the first data sharing consortium of clinical study sponsors and funders. CSDR is a leader in the data sharing community inspired to drive scientific innovation and improve medical care by facilitating access to patient-level data from clinical studies (https://www.novartisclinicaltrials.com/TrialConnectWeb/voluntarydataviewmore.nov).

Novartis is committed to sharing, upon requests from qualified external researchers and subsequent approval by an independent review panel based on scientific merit, anonymized patient-level and study-level clinical-trial data, and redacted clinical study reports, for medicines and indications approved in the United States and Europe after the respective study is accepted for publication. All data provided are anonymized to respect the privacy of patients who have participated in the trial in line with applicable laws and regulations. This trial data availability is according to the criteria and process described on www.clinicalstudydatarequest.com.
