# Supplementary material for: Single-Dose Intrathecal Dorsal Root Ganglia Toxicity of Onasemnogene Abeparvovec in Cynomolgus Monkeys
Source: Hum Gene Ther. 2022 Jul 13;33(13-14):740–56. doi: 10.1089/hum.2021.255 (PMC9347375; doi:10.1089/hum.2021.255)
Supplement: Supplemental data [file Suppl_TableS1.docx]

Supplemental Table 1. Study design and immunosuppressive regimens for the 13-week mechanistic study

| Group number | Test item | Dose concentration (vg/animal in 0.8 mL volume)^a^ | Dose concentration (mg/kg) | Animals/group | | Necropsy after | |
| --- | --- | --- | --- | --- | --- | --- | --- |
|  |  |  |  | Males | Females | 2 Weeks  (interim)^b^ | 13 Weeks (terminal)^c^ |
| 1^d^ | Vehicle | 0 | 0 | 5 | 5 | 3M/3F | 2M/2F |
| 2 | Onasemnogene abeparvovec | 3×10^13^ | – | 5 | 5 | 3M/3F | 2M/2F |
| 3^e^ | Onasemnogene abeparvovec + prednisolone | 3×10^13^ | –  1 | 5 | 5 | 3M/3F | 2M/2F |
| 4^f^ | Onasemnogene abeparvovec + rituximab (diphenhydramine)^g^ and everolimus | 3×10^13^ | – 20 4 0.5 | 5 | 5 | 3M/3F | 2M/2F |
| F, Females; M, Males; vg, vector genomes  ^a^Contrast agent was administered at a dose volume of 0.2 mL prior to onasemnogene abeparvovec or vehicle administration.  ^b^Animals designated for interim sacrifice underwent 2 weeks of observation following dose administration.  ^c^Animals designated for the terminal sacrifice underwent 13 weeks of observation following dose administration.  ^d^Group 1 was administered vehicle control item and contrast agent only.  ^e^Group 3 received prednisolone (1 mg/kg) by oral gavage beginning on the day before onasemnogene abeparvovec administration (Day –1) to Day 29 and then on Days 31, 33, 35, 37, 39, and 41 post-dose.  ^f^Group 4 received intravenous rituximab at 20 mg/kg 2 weeks before onasemnogene abeparvovec administration (Day –14) and every 14 days thereafter until Week 12, post-intrathecal injection, and received everolimus by oral gavage at 0.5 mg/kg once daily, beginning 2 weeks (Day –14) before onasemnogene abeparvovec administration and continuing until Week 2.  ^g^Diphenhydramine is an antihistamine, which was administered as a premedication to rituximab. | | | | | | | |
