# Supplementary material for: Single-Dose Intrathecal Dorsal Root Ganglia Toxicity of Onasemnogene Abeparvovec in Cynomolgus Monkeys
Source: Hum Gene Ther. 2022 Jul 13;33(13-14):740–56. doi: 10.1089/hum.2021.255 (PMC9347375; doi:10.1089/hum.2021.255)
Supplement: Supplemental data [file Suppl_TableS2.docx]

**Supplemental Table 2. Samples collected for histopathology analysis**

| Organ/tissue | 13-week intrathecal study | 12-month intrathecal GLP study | 6-month intravenous study |  |
| --- | --- | --- | --- | --- |
| Adrenals | X | X |  |  |
| Aorta | X | X |  |  |
| Bone, femur with bone marrow (articular surface of the distal end to include stifle joint) |  | X |  |  |
| Bone, sternum with bone marrow |  | X |  |  |
| Brain | X | X^a,b^ | X^a,b^ |  |
| Cecum | X | X |  |  |
| Cervix |  | X |  |  |
| Colon | X | X |  |  |
| Cranial (superior) cervical ganglia | X | X^b^ | X^b^ |  |
| Dorsal root ganglion (cervical) to include dorsal spinal root | X | X^a,b,c^ | X^b,d^ |  |
| Dorsal root ganglion (thoracic) to include dorsal spinal root | X | X^a,b,c^ | X^b,d^ |  |
| Dorsal root ganglion (lumbar) to include dorsal spinal root | X | X^a,b,c^ | X^b,d^ |  |
| Dorsal root ganglion (sacral) to include dorsal spinal root | X | X | X^b,d^ |  |
| Duodenum | X | X |  |  |
| Epididymes | X | X |  |  |
| Esophagus | X | X |  |  |
| Eyes | X | X^e^ |  |  |
| Fibular nerve | X^f^ |  | X^b,g^ |  |
| Gall bladder (drained) | X | X |  |  |
| Gut associated lymphoid tissue (GALT)/Peyer’s patch | X | X | X |  |
| Heart | X | X | X |  |
| Ileum | X | X |  |  |
| Jejunum | X | X |  |  |
| Kidneys | X | X | X |  |
| Lesions | X | X | X |  |
| Liver | X | X | X |  |
| Lungs with large bronchi | X | X | X |  |
| Lymph nodes, deep cervical | X |  |  |  |
| Lymph nodes, internal iliac | X |  |  |  |
| Lymph nodes, mandibular | X | X |  |  |
| Lymph nodes, mesenteric | X | X |  |  |
| Mammary gland (males and females) |  | X^h^ |  |  |
| Medial plantar nerve | X | X^b,g,i^ | X^b,g^ |  |
| Median nerve |  |  | X^b,g^ |  |
| Muscle, biceps femoris | X | X | X |  |
| Muscle, diaphragm |  | X | X |  |
| Optic nerves | X | X^e,g^ |  |  |
| Ovaries | X | X |  |  |
| Pancreas | X | X |  |  |
| Pituitary gland | X | X |  |  |
| Prostate | X | X |  |  |
| Radial nerve | X^f^ | X^b,g,i^ | X^b,g^ |  |
| Rectum | X | X |  |  |
| Salivary glands, mandibular | X | X |  |  |
| Sciatic nerve | X | X^b,g^ | X^b,g^ |  |
| Seminal vesicle | X | X |  |  |
| Skin/subcutis | X | X |  |  |
| Spinal cord (cervical, thoracic, and lumbar to include injection site) | X^j,k^ | X^a,b,l^ | X^b,m^ |  |
| Spinal cord, spinal nerve roots/cauda equina (3 transverse sections) |  | X^b^ |  |  |
| Spleen | X | X |  |  |
| Sternum with bone marrow | X |  |  |  |
| Stomach | X | X |  |  |
| Sural nerve | X^f^ | X^b,g^ | X^b,g^ |  |
| Testes | X | X^n^ |  |  |
| Thymus | X | X |  |  |
| Thyroid with parathyroids | X | X |  |  |
| Tibial nerve | X^f^ | X^b,g^ | X^b,g^ |  |
| Tongue | X | X |  |  |
| Trachea | X | X |  |  |
| Trigeminal ganglion | X | X^b^ | X^b^ |  |
| Ulnar nerve | X^f^ | X^b,g,i^ | X^b,g^ |  |
| Urinary bladder | X | X |  |  |
| Uterus | X | X |  |  |
| Vagina |  | X |  |  |

^a^See Special Procedures for trimming directive.

^b^Collected in 10% neutral-buffered formalin for at least two but no more than three overnights and then processed to paraffin block.

^c^Collect at least n=6 each from cervical and thoracic regions, n=6 each from lumbar region, and at least n=2 from sacral regions.

^d^At least n=6 each from cervical, thoracic, and lumbar regions, and at least n=3 from sacral regions were collected.

^e^Collected in modified Davidson’s for at least two but no more than three overnights and then processed to paraffin block.

^f^Collected as distal as possible, unilateral, cross, longitudinal.

^g^Prepare transverse and longitudinal sections.

^h^Recuts not needed for males.

^i^Collected at the terminal necropsy.

^j^Stored in Actril^®^ and sent to the supplier for refurbishment. The location of the catheter tip was documented.

^k^Lumbosacral spinal cord at area of injection site (spinal column L4/5, vertebrae L1/2, or as documented in study) collect, trim and process n=6 transverse section in the region of the injection site.

^l^Spinal cord (cervical, thoracic, lumbar) prepared transverse and vertical oblique (after Day 91) or oblique (before Day 91) sections and place each region in a separate cassette. Lumbar spinal cord at area of injection site (spinal column L4/5, vertebrae L1/2 or as documented in study) collect, trim, and process n=6 transverse sections in the region of the injection site in addition to transverse and oblique sections.

^m^Spinal cord (cervical, thoracic, lumbar) prepared transverse and vertical oblique sections and each region placed in a separate cassette/block/slide.

^n^Collected in modified Davidson’s fixative and stored in 10% neutral‑buffered formalin.
