# Supplementary material for: Single-Dose Intrathecal Dorsal Root Ganglia Toxicity of Onasemnogene Abeparvovec in Cynomolgus Monkeys
Source: Hum Gene Ther. 2022 Jul 13;33(13-14):740–56. doi: 10.1089/hum.2021.255 (PMC9347375; doi:10.1089/hum.2021.255)
Supplement: Supplemental data [file Suppl_TableS3-S5.docx]

**Supplemental Table 3. Pre-study serum anti-AAV9 antibody titers in the 12-month GLP study.**

| **Dose Group** | **Dose** | **Animal ID** | **Titer** |
| --- | --- | --- | --- |
| **Group 1^a^** | Control | P0001 | 1:320 |
|  |  | P0002 | 1:640 |
|  |  | P0003 | <1:20 |
|  |  | P0004 | <1:20 |
|  |  | P0005 | 1:20,480 |
|  |  | P0401 | 1:160 |
|  |  | P0402 | 1:2,560 |
|  |  | P0403 | 1:80 |
|  |  | P0404 | <1:20 |
|  |  | P0405 | 1:80 |
| **Group 2^b^** | Onasemnogene abeparvovec (low dose) | P0101 | 1:80 |
|  |  | P0102 | 1:80 |
|  |  | P0103 | <1:20 |
|  |  | P0104 | <1:20 |
|  |  | P0105 | 1:20 |
|  |  | P0501 | <1:20 |
|  |  | P0502 | 1:80 |
|  |  | P0503 | <1:20 |
|  |  | P0504 | <1:20 |
|  |  | P0505 | 1:160 |
| **Group 3^c^** | Onasemnogene abeparvovec (mid dose) | P0201 | 1:40 |
|  |  | P0202 | 1:40 |
|  |  | P0203 | 1:80 |
|  |  | P0204 | <1:20 |
|  |  | P0205 | 1:40 |
|  |  | P0601 | 1:160 |
|  |  | P0602 | <1:20 |
|  |  | P0603 | <1:20 |
|  |  | P0604 | 1:1,280 |
|  |  | P0605 | 1:2,560 |
|  |  | P0301 | 1:80 |
| **Group 4^d^** | Onasemnogene abeparvovec (high dose) | P0302 | <1:20 |
|  |  | P0303 | <1:20 |
|  |  | P0304 | <1:20 |
|  |  | P0305 | 1:80 |
|  |  | P0701 | <1:20 |
|  |  | P0702 | <1:20 |
|  |  | P0703 | 1:80 |
|  |  | P0704 | <1:20 |
|  |  | P0705 | 1:20 |

^a^Group 1 received a single dose of vehicle control article.

^b^Group 2 received a single dose of 1.2×10^13^ vg/animal onasemnogene abeparvovec.

^c^Group 3 received a single dose of 3.0×10^13^ vg/animal onasemnogene abeparvovec.

^d^Group 4 received a single dose of 6.0×10^13^ vg/animal onasemnogene abeparvovec.

**Supplemental Table 4. Pre-study serum anti-AAV9 antibody titers in the 13-week mechanistic study.**

| **Dose Group** | **Dose** | **Animal ID** | **Titer** |
| --- | --- | --- | --- |
| **Group 1^a^** | Control | P0001 (M) | 3.15 |
|  |  | P0002 (M) | 3.76 |
|  |  | P0003 (M) | 3.34 |
|  |  | P0004 (M) | 4.67 |
|  |  | P0005 (M) | 5.44 |
|  |  | P0401 (F) | 3.08 |
|  |  | P0402 (F) | 4.19 |
|  |  | P0403 (F) | 4.66 |
|  |  | P0404 (F) | 4.64 |
|  |  | P0405 (F) | NQ |
| **Group 2^b^** | Onasemnogene abeparvovec | P0101 (M) | 3.8 |
|  |  | P0102 (M) | 4.66 |
|  |  | P0103 (M) | 2.36 |
|  |  | P0104 (M) | 5.05 |
|  |  | P0105 (M) | 3.45 |
|  |  | P0501 (F) | 3.02 |
|  |  | P0502 (F) | NQ |
|  |  | P0503 (F) | 3.77 |
|  |  | P0504 (F) | 2.67 |
|  |  | P0505 (F) | 2.27 |
| **Group 3^c^** | Onasemnogene abeparvovec + prednisolone | P0201 (M) | NQ |
|  |  | P0202 (M) | 3.61 |
|  |  | P0203 (M) | NQ |
|  |  | P0204 (M) | 1.88 |
|  |  | P0205 (M) | 1.77 |
|  |  | P0601 (F) | 3.02 |
|  |  | P0602 (F) | 3.87 |
|  |  | P0603 (F) | 3.91 |
|  |  | P0604 (F) | 3.12 |
|  |  | P0605 (F) | 4.66 |
| **Group 4^d^** | Onasemnogene abeparvovec + rituximab + everolimus | P0301 (M) | NQ |
|  |  | P0302 (M) | NQ |
|  |  | P0303 (M) | 2.38 |
|  |  | P0304 (M) | 2.15 |
|  |  | P0305 (M) | 2.48 |
|  |  | P0701 (F) | NQ |
|  |  | P0702 (F) | 4.27 |
|  |  | P0703 (F) | 3.15 |
|  |  | P0704 (F) | 3.05 |
|  |  | P0705 (F) | 2.92 |

AAV9, adeno-associated virus serotype 9; NQ, not quantifiable (signals below cut point).

^a^Group 1 was administered vehicle control item and contrast agent only.

^b^Group 2 was administered onasemnogene abeparvovec.

^c^Group 3 received prednisolone (1 mg/kg) by oral gavage beginning on the day before onasemnogene abeparvovec administration (Day –1) to Day 29 and then on Days 31, 33, 35, 37, 39, and 41 post-dose.

^d^Group 4 received intravenous rituximab at 20 mg/kg 2 weeks before onasemnogene abeparvovec administration (Day –14) and every 14 days thereafter until Week 12, post-intrathecal injection, and received everolimus by oral gavage at 0.5 mg/kg once daily, beginning 2 weeks (Day –14) before onasemnogene abeparvovec administration and continuing until Week 2.

**Supplemental Table 5. Pre-study serum anti-AAV9 antibody titers in the 6-month intravenous GLP study.**

| **Dose Group** | **Dose** | **Animal ID** | **Titer** |
| --- | --- | --- | --- |
| **Group 1^a^** | Control | P0001 | 1:160 |
|  |  | P0002 | 1:160 |
|  |  | P0003 | <1:20 |
|  |  | P0004 | 1:20,480 |
|  |  | P0005 | <1:20 |
|  |  | P0006 | 1:320 |
|  |  | P0301 | <1:20 |
|  |  | P0302 | 1:160 |
|  |  | P0303 | 1:640 |
|  |  | P0304 | <1:20 |
|  |  | P0305 | <1:20 |
|  |  | P0306 | <1:20 |
| **Group 2^b^** | Onasemnogene abeparvovec | P0101 | 1:1,280 |
|  |  | P0102 | 1:320 |
|  |  | P0103 | 1:10,240 |
|  |  | P0104 | 1:5,120 |
|  |  | P0105 | 1:640 |
|  |  | P0106 | 1:20 |
|  |  | P0401 | <1:20 |
|  |  | P0402 | <1:20 |
|  |  | P0403 | <1:20 |
|  |  | P0404 | <1:20 |
|  |  | P0405 | 1:20 |
|  |  | P0406 | <1:20 |
| **Group 3^c^** | Onasemnogene abeparvovec + prednisolone | P0201 | <1:20 |
|  |  | P0202 | <1:20 |
|  |  | P0203 | 1:80 |
|  |  | P0204 | 1:1,280 |
|  |  | P0205 | 1:80 |
|  |  | P0206 | <1:20 |
|  |  | P0501 | 1:20 |
|  |  | P0502 | 1:160 |
|  |  | P0503 | <1:20 |
|  |  | P0504 | 1:80 |
|  |  | P0505 | 1:40 |
|  |  | P0506 | <1:20 |

^a^Group 1 was administered vehicle control article and placebo.

^b^Group 2 was administered 1.1×10^14^ vg/kg onasemnogene abeparvovec (the approved clinical dose) and placebo.

^c^Group 3 was administered 1.1×10^14^ vg/kg and prednisolone.
