# Supplementary material for: Single-Dose Intrathecal Dorsal Root Ganglia Toxicity of Onasemnogene Abeparvovec in Cynomolgus Monkeys
Source: Hum Gene Ther. 2022 Jul 13;33(13-14):740–56. doi: 10.1089/hum.2021.255 (PMC9347375; doi:10.1089/hum.2021.255)
Supplement: Supplemental data [file Suppl_TableS6-S8.docx]

**Supplemental Table 6. Onasemnogene abeparvovec DNA concentrations in the DRG at 6 and 52 weeks of observation post-intrathecal dosing in the 12-month GLP study.**

| **Onasemnogene abeparvovec DNA concentrations in the DRG at 6 weeks post-dose** | | | | | | | | | | |
| --- | --- | --- | --- | --- | --- | --- | --- | --- | --- | --- |
|  | **Group 1 (Control)^a^** | | | | | | | | | |
|  | P0001 | | P0002 | | P0003 | P0401 | | P0402 | | P0403 |
| Cervical DRG | BLQ | | BLQ | | BLQ | BLQ | | BLQ | | BLQ |
| Thoracic DRG | BLQ | | BLQ | | BLQ | BLQ | | BLQ | | BLQ |
| Lumbar DRG | BLQ | | BLQ | | BLQ | BLQ | | BLQ | | BLQ |
| Sacral DRG | BLQ | | BLQ | | BLQ | BLQ | | BLQ | | BLQ |
|  | **Group 2 (onasemnogene abeparvovec low dose)^b^** | | | | | | | | | |
|  | P0101 | | P0102 | | P0103 | P0501 | | P0502 | | P0503 |
| Cervical DRG | 5.18 | | 13.3 | | 11.1 | 0.653 | | 0.364 | | 2.91 |
| Thoracic DRG | 3.69 | | 5.17 | | 1.40 | 8.71 | | 0.453 | | 5.3 |
| Lumbar DRG | 0.657 | | 2.87 | | 0.767 | 1.48 | | 1.07 | | 2.13 |
| Sacral DRG | 2.41 | | 8.53 | | 1.77 | 3.23 | | 6.98 | | 3.73 |
|  | **Group 3 (onasemnogene abeparvovec mid dose)^c^** | | | | | | | | | |
|  | P0201 | | P0202 | | P0203 | P0601 | | P0602 | | P0603 |
| Cervical DRG | 3.11 | | 1.27 | | 5.82 | 1.08 | | 1.36 | | 9.27 |
| Thoracic DRG | 1.45 | | 2.32 | | 7.60 | 0.867 | | 8.06 | | 1.32 |
| Lumbar DRG | 4.02 | | 2.93 | | 2.85 | 1.22 | | 0.750 | | 2.32 |
| Sacral DRG | 12.1 | | 2.82 | | 13.4 | 0.697 | | 1.46 | | 1.22 |
|  | **Group 4 (onasemnogene abeparvovec high dose)^d^** | | | | | | | | | |
|  | P0301 | | P0302 | | P0303 | P0701 | | P0702 | | P0703 |
| Cervical DRG | 1.77 | | 1.65 | | 27.4 | 2.21 | | 1.80 | | 5.52 |
| Thoracic DRG | 2.35 | | 4.00 | | 1.75 | 4.47 | | 3.54 | | 6.61 |
| Lumbar DRG | 4.15 | | 1.84 | | 2.73 | 0.592 | | 3.13 | | 1.36 |
| Sacral DRG | 3.54 | | 6.82 | | 7.87 | 4.81 | | 45.4 | | 60.7 |
| **Onasemnogene abeparvovec DNA concentrations in the DRG at 52 weeks post-dose** | | | | | | | | | | |
|  | | **Group 1 (Control)^a^** | | | | | | | | |
|  | | P0004 | | P0005 | | | P0404 | | P0405 | |
| Cervical DRG | | BLQ | | BLQ | | | BLQ | | BLQ | |
| Thoracic DRG | | BLQ | | BLQ | | | BLQ | | BLQ | |
| Lumbar DRG | | BLQ | | BLQ | | | BLQ | | BLQ | |
| Sacral DRG | | BLQ | | BLQ | | | BLQ | | BLQ | |
|  | | **Group 2 (onasemnogene abeparvovec low dose)^b^** | | | | | | | | |
|  | | P0104 | | P0105 | | | P0504 | | P0505 | |
| Cervical DRG | | 0.216 | | 0.885 | | | 0.786 | | 0.497 | |
| Thoracic DRG | | 0.206 | | 2.55 | | | 2.57 | | 0.862 | |
| Lumbar DRG | | 1.36 | | 0.343 | | | 1.33 | | 0.283 | |
| Sacral DRG | | 1.89 | | 1.59 | | | 1.62 | | 0.067 | |
|  | | **Group 3 (onasemnogene abeparvovec mid dose)^c^** | | | | | | | | |
|  | | P0204 | | P0205 | | | P0604 | | P0605 | |
| Cervical DRG | | 0.116 | | 0.300 | | | 0.556 | | 0.670 | |
| Thoracic DRG | | 1.67 | | 0.909 | | | 17.1 | | 2.88 | |
| Lumbar DRG | | 1.94 | | 2.39 | | | 0.908 | | 2.52 | |
| Sacral DRG | | 0.666 | | 14.6 | | | 7.87 | | 1.72 | |
|  | | **Group 4 (onasemnogene abeparvovec high dose)^d^** | | | | | | | | |
|  | | P0304 | | P0305 | | | P0704 | | P0705 | |
| Cervical DRG | | 0.932 | | 11.4 | | | 0.843 | | 1.00 | |
| Thoracic DRG | | 2.16 | | 1.25 | | | 3.41 | | 2.30 | |
| Lumbar DRG | | 2.72 | | 2.56 | | | 2.15 | | 2.83 | |
| Sacral DRG | | 3.39 | | 4.48 | | | 3.54 | | 22.5 | |

BLQ, below lower limit of quantitation (28 copies/reaction); DRG, dorsal root ganglion; GLP, Good Laboratory Practice.

^a^Group 1 received a single dose of vehicle control article.

^b^Group 2 received a single dose of 1.2×10^13^ vg/animal onasemnogene abeparvovec.

^c^Group 3 received a single dose of 3.0×10^13^ vg/animal onasemnogene abeparvovec.

^d^Group 4 received a single dose of 6.0×10^13^ vg/animal onasemnogene abeparvovec.

**Supplemental Table 7. Onasemnogene abeparvovec DNA concentrations in the DRG at 2 and 13 weeks of observation post-intrathecal dosing in the 13-week mechanistic study.**

| **Onasemnogene abeparvovec DNA concentrations in the DRG at 2 weeks post-dose** | | | | | | | | | | |
| --- | --- | --- | --- | --- | --- | --- | --- | --- | --- | --- |
|  | **Group 1 (Control)^a^** | | | | | | | | | |
|  | P0001 | | P0002 | | P0003 | P0403 | | P0404 | | P0405 |
| Cervical DRG | BLQ | | BLQ | | BLQ | BLQ | | BLQ | | BLQ |
| Thoracic DRG | BLQ | | BLQ | | BLQ | BLQ | | BLQ | | BLQ |
| Lumbar DRG | BLQ | | BLQ | | BLQ | BLQ | | BLQ | | BLQ |
| Sacral DRG | BLQ | | BLQ | | BLQ | BLQ | | BLQ | | BLQ |
|  | **Group 2 (onasemnogene abeparvovec)^b^** | | | | | | | | | |
|  | P0101 | | P0102 | | P0103 | P0501 | | P0502 | | P0503 |
| Cervical DRG | 0.971 | | 0.288 | | 0.647 | 1.18 | | BLQ | | BLQ |
| Thoracic DRG | 0.522 | | 4.05 | | 0.330 | 2.02 | | 5.57 | | 0.119 |
| Lumbar DRG | 1.52 | | 11.6 | | 0.379 | 0.226 | | 9.81 | | 2.83 |
| Sacral DRG | 30.4 | | 37.4 | | 0.730 | 6.95 | | BLQ | | 8.75 |
|  | **Group 3 (onasemnogene abeparvovec + prednisolone)^c^** | | | | | | | | | |
|  | P0201 | | P0202 | | P0203 | P0603 | | P0604 | | P0605 |
| Cervical DRG | BLQ | | BLQ | | 0.0700 | 0.0258 | | 0.0896 | | 0.230 |
| Thoracic DRG | 0.334 | | 7.51 | | 5.41 | BLQ | | 2.57 | | 0.306 |
| Lumbar DRG | 0.579 | | 2.88 | | 2.18 | 0.352 | | 0.411 | | 0.715 |
| Sacral DRG | 1.35 | | 3.52 | | 10.306 | 50.9 | | 24.6 | | 0.472 |
|  | **Group 4 (onasemnogene abeparvovec + rituximab + everolimus)^d^** | | | | | | | | | |
|  | P0301 | | P0302 | | P0303 | P0701 | | P0702 | | P0703 |
| Cervical DRG | 0.143 | | 0.361 | | 0.555 | 0.584 | | BLQ | | BLQ |
| Thoracic DRG | 0.312 | | 1.55 | | 2.16 | 2.45 | | 1.02 | | 0.591 |
| Lumbar DRG | 1.59 | | 2.73 | | 1.59 | 1.18 | | 0.458 | | 0.726 |
| Sacral DRG | 1.03 | | 0.815 | | 150 | 12.4 | | 2.06 | | 0.817 |
| **Onasemnogene abeparvovec DNA concentrations in the DRG at 13 weeks post-dose** | | | | | | | | | | |
|  | | **Group 1 (Control)^a^** | | | | | | | | |
|  | | P0004 | | P0005 | | | P0401 | | P0402 | |
| Cervical DRG | | BLQ | | BLQ | | | BLQ | | BLQ | |
| Thoracic DRG | | BLQ | | BLQ | | | BLQ | | BLQ | |
| Lumbar DRG | | BLQ | | BLQ | | | BLQ | | BLQ | |
| Sacral DRG | | BLQ | | BLQ | | | BLQ | | BLQ | |
|  | | **Group 2 (onasemnogene abeparvovec)^b^** | | | | | | | | |
|  | | P0104 | | P0105 | | | P0504 | | P0505 | |
| Cervical DRG | | 1.82 | | 0.447 | | | 0.457 | | BLQ | |
| Thoracic DRG | | 0.514 | | 4.01 | | | 0.246 | | 0.0733 | |
| Lumbar DRG | | 0.615 | | 0.779 | | | 4.60 | | 0.364 | |
| Sacral DRG | | 1.17 | | NS | | | 22.2 | | 8.54 | |
|  | | **Group 3 (onasemnogene abeparvovec + prednisolone)^c^** | | | | | | | | |
|  | | P0204 | | P0205 | | | P0601 | | P0602 | |
| Cervical DRG | | BLQ | | BLQ | | | BLQ | | BLQ | |
| Thoracic DRG | | 2.21 | | 0.809 | | | 0.648 | | BLQ | |
| Lumbar DRG | | 0.373 | | 0.849 | | | 0.696 | | 0.206 | |
| Sacral DRG | | 1.20 | | NS | | | 2.38 | | 0.100 | |
|  | | **Group 4 (onasemnogene abeparvovec + rituximab + everolimus)^d^** | | | | | | | | |
|  | | P0304 | | P0305 | | | P0704 | | P0705 | |
| Cervical DRG | | BLQ | | 0.0476 | | | BLQ | | 0.665 | |
| Thoracic DRG | | 2.87 | | 0.866 | | | 2.07 | | 0.789 | |
| Lumbar DRG | | 1.05 | | 0.785 | | | 1.20 | | 12.7 | |
| Sacral DRG | | 35.0 | | 2.17 | | | 17.6 | | 114 | |

BLQ, below lower limit of quantitation (28 copies/reaction); DRG, dorsal root ganglion; NS, no sample collected.

^a^Group 1 was administered vehicle control item and contrast agent only.

^b^Group 2 was administered onasemnogene abeparvovec.

^c^Group 3 received prednisolone (1 mg/kg) by oral gavage beginning on the day before onasemnogene abeparvovec administration (Day –1) to Day 29 and then on Days 31, 33, 35, 37, 39, and 41 post-dose.

^d^Group 4 received intravenous rituximab at 20 mg/kg 2 weeks before onasemnogene abeparvovec administration (Day –14) and every 14 days thereafter until Week 12, post-intrathecal injection, and received everolimus by oral gavage at 0.5 mg/kg once daily, beginning 2 weeks (Day –14) before onasemnogene abeparvovec administration and continuing until Week 2.

**Supplemental Table 8. Onasemnogene abeparvovec DNA concentrations in the DRG at 6 weeks and 6 months of observation post-intravenous dosing in the 6-month intravenous GLP study.**

| **Onasemnogene abeparvovec DNA concentrations in the DRG at 6 weeks post-dose** | | | | | | |
| --- | --- | --- | --- | --- | --- | --- |
|  | **Group 1 (Control)^a^** | | | | | |
|  | P0001 | P0002 | P0003 | P0301 | P0302 | P0303 |
| Cervical DRG | BLQ | BLQ | BLQ | BLQ | BLQ | BLQ |
| Thoracic DRG | BLQ | BLQ | BLQ | BLQ | BLQ | BLQ |
| Lumbar DRG | BLQ | BLQ | BLQ | BLQ | BLQ | BLQ |
| Sacral DRG | BLQ | BLQ | BLQ | BLQ | BLQ | BLQ |
|  | **Group 2 (onasemnogene abeparvovec)^b^** | | | | | |
|  | P0101 | P0102 | P0103 | P0401 | P0402 | P0403 |
| Cervical DRG | 0.265 | 0.692 | 0.113 | 0.905 | 6.17 | 1.09 |
| Thoracic DRG | 0.327 | 0.988 | 0.117 | 0.823 | 60.3 | 1.49 |
| Lumbar DRG | 0.316 | 1.19 | 0.0961 | 1.24 | 0.541 | 1.18 |
| Sacral DRG | 0.405 | 1.15 | 0.111 | 1.50 | 0.702 | 1.96 |
|  | **Group 3 (onasemnogene abeparvovec + prednisolone)^c^** | | | | | |
|  | P0201 | P0202 | P0203 | P0501 | P0502 | P0503 |
| Cervical DRG | 1.06 | 0.732 | 0.883 | 0.752 | 0.269 | 0.495 |
| Thoracic DRG | 5.89 | 0.703 | 4.03 | 1.66 | 0.424 | 11.7 |
| Lumbar DRG | 2.04 | 1.04 | 1.39 | 1.47 | 0.706 | 1.02 |
| Sacral DRG | 3.17 | 1.12 | 2.04 | 0.691 | 0.404 | 1.49 |
| **Onasemnogene abeparvovec DNA concentrations in the DRG at 6 months post-dose** | | | | | | |
|  | **Group 1 (Control)^a^** | | | | | |
|  | P0004 | P0005 | P0006 | P0304 | P0305 | P0306 |
| Cervical DRG | BLQ | BLQ | BLQ | BLQ | BLQ | BLQ |
| Thoracic DRG | BLQ | BLQ | BLQ | BLQ | BLQ | BLQ |
| Lumbar DRG | BLQ | BLQ | BLQ | BLQ | BLQ | BLQ |
| Sacral DRG | BLQ | BLQ | BLQ | BLQ | BLQ | BLQ |
|  | **Group 2 (onasemnogene abeparvovec)^b^** | | | | | |
|  | P0104 | P0105 | P0106 | P0404 | P0405 | P0406 |
| Cervical DRG | 0.304 | 0.0534 | 0.309 | 0.485 | 0.346 | 1.17 |
| Thoracic DRG | 0.383 | 1.38 | 0.724 | 0.770 | 0.324 | 1.49 |
| Lumbar DRG | 0.713 | 0.0964 | 0.553 | 0.857 | 0.455 | 1.64 |
| Sacral DRG | 0.535 | 0.0638 | 0.582 | 0.776 | 0.388 | 1.91 |
|  | **Group 3 (onasemnogene abeparvovec + prednisolone)^c^** | | | | | |
|  | P0204 | P0205 | P0206 | P0504 | P0505 | P0506 |
| Cervical DRG | 0.0102 | 0.280 | 0.494 | 0.490 | 0.336 | 1.01 |
| Thoracic DRG | BLQ | 0.420 | 0.682 | 0.566 | 1.06 | 0.771 |
| Lumbar DRG | BLQ | 0.807 | 1.26 | 0.732 | 0.563 | 1.14 |
| Sacral DRG | BLQ | 0.632 | 1.42 | 0.867 | 0.745 | 1.36 |

BLQ, below lower limit of quantitation (28 copies/reaction); DRG, dorsal root ganglion; GLP, Good Laboratory Practice.

^a^Group 1 was administered vehicle control article and placebo.

^b^Group 2 was administered 1.1×10^14^ vg/kg onasemnogene abeparvovec (the approved clinical dose) and placebo.

^c^Group 3 was administered 1.1×10^14^ vg/kg and prednisolone.
