# Supplementary material for: Single-Dose Intrathecal Dorsal Root Ganglia Toxicity of Onasemnogene Abeparvovec in Cynomolgus Monkeys
Source: Hum Gene Ther. 2022 Jul 13;33(13-14):740–56. doi: 10.1089/hum.2021.255 (PMC9347375; doi:10.1089/hum.2021.255)
Supplement: Supplemental data [file Suppl_TableS9.docx]

**Supplemental Table 9. Immunosuppressant-related CSF effects observed in the 13-week mechanistic study**

|  | Onasemnogene abeparvovec | | | | | |
| --- | --- | --- | --- | --- | --- | --- |
| Sex | Males | | | Females | | |
| Dose (vg/animal) | 3×10^13^ | | | 3×10^13^ | | |
| Group | 2 | 3 | 4 | 2 | 3 | 4 |
| Immunosuppressant(s) | None | Prednisolone | Rituximab+ everolimus | None | Prednisolone | Rituximab+ everolimus |
| Dose (mg/kg) | None | 1 | 20+0.5 | None | 1 | 20+0.5 |
| Total protein concentration |  |  |  |  |  |  |
| Dosing Day 15 | – | – | – | – | – | – |
| Dosing Day 92 | – | – | – | +281.0%^a^ | +278.8%^b^ | +390.2%^c^ |
| Microalbumin concentration |  | – |  |  |  |  |
| Dosing Day 15 | – | – | – | – | – | – |
| Dosing Day 92 | – | – | – | +272.8%^a^ | +306.6%^b^ | +436.7%^c^ |
| – = No effect observed; + = Increase.  Note: Values for onasemnogene abeparvovec-dosed groups are expressed as a % difference from the pre-dose phase individual value.  ^a^Value for Animal P0504.  ^b^Value for Animal P0601.  ^c^Value for Animal P0705. | | | | | | |
