# Supplementary material for: Single-Dose Intrathecal Dorsal Root Ganglia Toxicity of Onasemnogene Abeparvovec in Cynomolgus Monkeys
Source: Hum Gene Ther. 2022 Jul 13;33(13-14):740–56. doi: 10.1089/hum.2021.255 (PMC9347375; doi:10.1089/hum.2021.255)
Supplement: Supplemental data [file Suppl_FigS2.docx]

**Supplemental Figure 2.** **Mean plasma concentrations of NfL in the 6-month intravenous GLP study.**

**
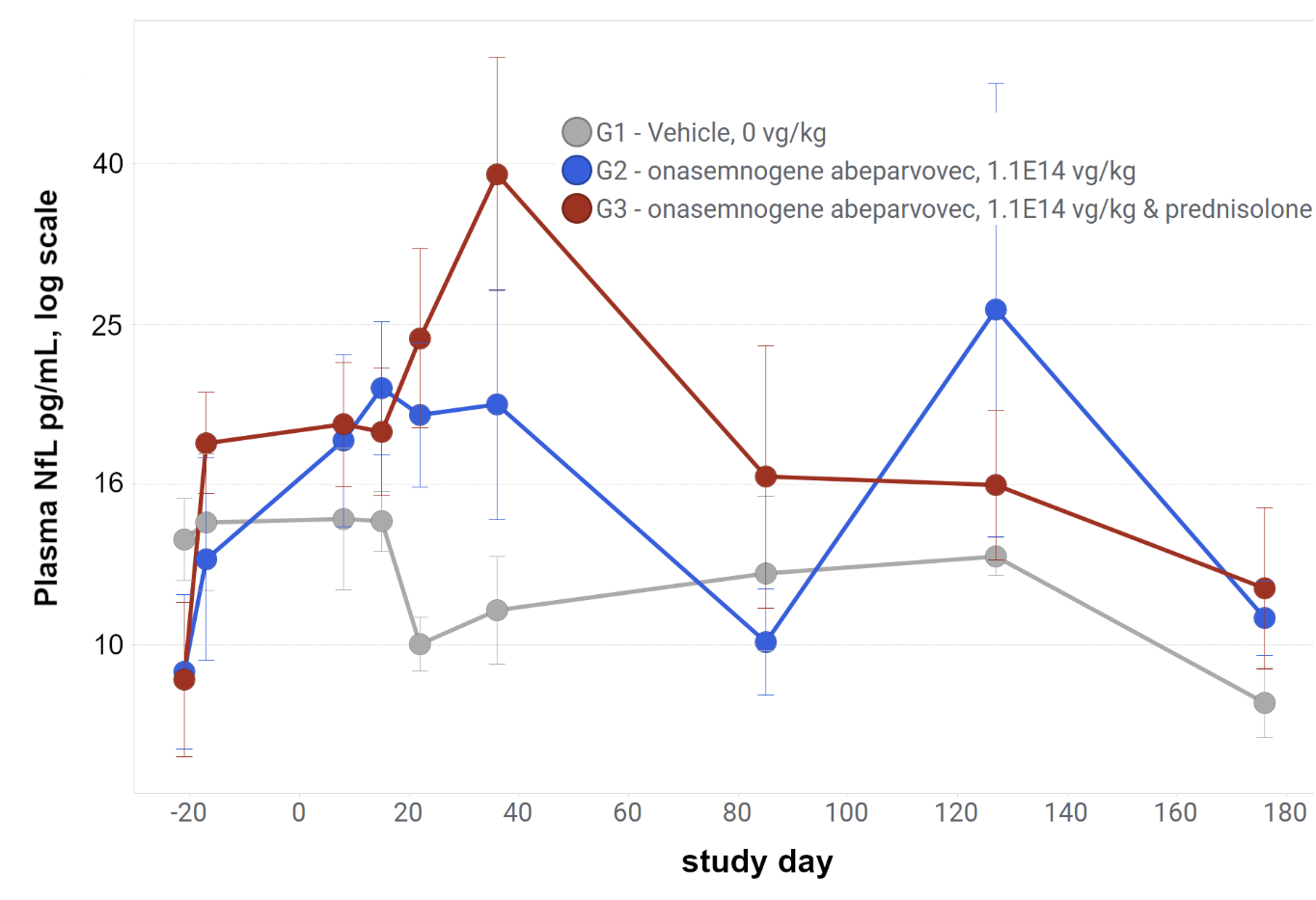
**

Plasma NfL concentrations following single-dose intrathecal administration of onasemnogene abeparvovec (1.1×10^14^ vg/kg) with or without co-administration of prednisolone.
