# Supplementary material for: Single-Dose Intrathecal Dorsal Root Ganglia Toxicity of Onasemnogene Abeparvovec in Cynomolgus Monkeys
Source: Hum Gene Ther. 2022 Jul 13;33(13-14):740–56. doi: 10.1089/hum.2021.255 (PMC9347375; doi:10.1089/hum.2021.255)
Supplement: Supplemental data [file Suppl_TableS10.docx]

**Supplemental Table 10. Effect of immunosuppression on onasemnogene abeparvovec–related dorsal root ganglion microscopic findings at 2 and 13 weeks of observation post-intrathecal dosing for males in the 13-week mechanistic study**

| Tissue/finding | Males | | | | | | | |
| --- | --- | --- | --- | --- | --- | --- | --- | --- |
| Dose (vg/animal) | 0 | 0 | 3×10^13^ | 3×10^13^ | 3×10^13^ | 3×10^13^ | 3×10^13^ | 3×10^13^ |
| Prednisolone (mg/kg) | 0 | 0 | 0 | 0 | 1 | 1 | 0 | 0 |
| Rituximab (mg/kg) | 0 | 0 | 0 | 0 | 0 | 0 | 20 | 20 |
| Diphenhydramine (mg/kg) | 0 | 0 | 0 | 0 | 0 | 0 | 4 | 4 |
| Everolimus (mg/kg) | 0 | 0 | 0 | 0 | 0 | 0 | 0.5 | 0.5 |
| Necropsy/Study Day | 15 | 92 | 15 | 92 | 15 | 92 | 15 | 92 |
| **Ganglion,** **cervical dorsal root** |  |  |  |  |  |  |  |  |
| Number examined | 3 | 2 | 3 | 2 | 3 | 2 | 3 | 2 |
| Inflammation, mononuclear cell |  |  |  |  |  |  |  |  |
| Total number affected | 0 | 0 | 1 | 0 | 1 | 1 | 1 | 1 |
| Minimal | 0 | 0 | 0 | 0 | 1 | 1 | 0 | 1 |
| Slight | 0 | 0 | 1 | 0 | 0 | 0 | 1 | 0 |
| Degeneration, neuron |  |  |  |  |  |  |  |  |
| Total number affected | 0 | 0 | 1 | 0 | 1 | 1 | 1 | 1 |
| Minimal | 0 | 0 | 0 | 0 | 1 | 1 | 0 | 1 |
| Slight | 0 | 0 | 1 | 0 | 0 | 0 | 1 | 0 |
| Degeneration, axon, spinal root/spinal nerve |  |  |  |  |  |  |  |  |
| Total number affected | 0 | 0 | 0 | 0 | 0 | 0 | 1 | 1 |
| Slight | 0 | 0 | 0 | 0 | 0 | 0 | 1 | 1 |
| Infiltrate, mononuclear cell, spinal root/spinal nerve |  |  |  |  |  |  |  |  |
| Total number affected | 0 | 0 | 0 | 1 | 0 | 1 | 0 | 1 |
| Minimal | 0 | 0 | 0 | 1 | 0 | 1 | 0 | 1 |
| Satellite glial cells, increased |  |  |  |  |  |  |  |  |
| Total number affected | 0 | N/A | 2 | N/A | 2 | N/A | 3 | N/A |
| Minimal | 0 | N/A | 2 | N/A | 2 | N/A | 3 | N/A |
| Satellite glial cell, increased/neuronal cell loss |  |  |  |  |  |  |  |  |
| Total number affected | N/A | 0 | N/A | 0 | N/A | 0 | N/A | 0 |
| Minimal | N/A | 0 | N/A | 0 | N/A | 0 | N/A | 0 |
| **Spinal cord,** c**ervical** |  |  |  |  |  |  |  |  |
| Number examined | 3 | 2 | 3 | 2 | 3 | 2 | 3 | 2 |
| Degeneration, axon, dorsal funiculus |  |  |  |  |  |  |  |  |
| Total number affected | 0 | 1 | 0 | 1 | 0 | 2 | 2 | 2 |
| Minimal | 0 | 0 | 0 | 0 | 0 | 1 | 2 | 0 |
| Slight | 0 | 1 | 0 | 0 | 0 | 1 | 0 | 2 |
| Moderate | 0 | 0 | 0 | 1 | 0 | 0 | 0 | 0 |

N/A, not available.
