# Supplementary material for: Single-Dose Intrathecal Dorsal Root Ganglia Toxicity of Onasemnogene Abeparvovec in Cynomolgus Monkeys
Source: Hum Gene Ther. 2022 Jul 13;33(13-14):740–56. doi: 10.1089/hum.2021.255 (PMC9347375; doi:10.1089/hum.2021.255)
Supplement: Supplemental data [file Suppl_TableS11.docx]

**Supplemental Table 11. Summary incidence and severity onasemnogene abeparvovec–related microscopic findings in the dorsal root ganglia and trigeminal ganglia** **at 6 and 52 weeks of observation post-intrathecal dosing for males in the 12-month GLP study**

| Tissue/finding |  | Males | | | | | | | |
| --- | --- | --- | --- | --- | --- | --- | --- | --- | --- |
| Dose (vg/animal in 0.80 mL volume) | | 0 | 0 | 1.2×10^13^ | 1.2×10^13^ | 3×10^13^ | 3×10^13^ | 6×10^13^ | 6×10^13^ |
| Number examined | | 3 | 2 | 3 | 2 | 3 | 2 | 3 | 2 |
| Necropsy Date (Study Day) | | 43 | 365 | 43 | 365 | 43 | 365 | 43 | 365 |
| **Ganglion, cervical dorsal root** | |  |  |  |  |  |  |  |  |
| Inflammation, mononuclear cell | |  |  |  |  |  |  |  |  |
| Total number affected | | 0 | 0 | 3 | 0 | 1 | 0 | 0 | 1 |
| Minimal | | 0 | 0 | 0 | 0 | 0 | 0 | 0 | 1 |
| Slight | | 0 | 0 | 1 | 0 | 1 | 0 | 0 | 0 |
| Moderate | | 0 | 0 | 2 | 0 | 0 | 0 | 0 | 0 |
| Degeneration, neuron | |  |  |  |  |  |  |  |  |
| Total number affected | | 0 | 0 | 3 | 0 | 1 | 0 | 1 | 0 |
| Minimal | | 0 | 0 | 0 | 0 | 0 | 0 | 0 | 0 |
| Slight | | 0 | 0 | 1 | 0 | 1 | 0 | 1 | 0 |
| Moderate | | 0 | 0 | 2 | 0 | 0 | 0 | 0 | 0 |
| Degeneration, axon,  spinal root/spinal nerve | |  |  |  |  |  |  |  |  |
| Total number affected | | 0 | 0 | 3 | 0 | 1 | 0 | 1 | 0 |
| Minimal | | 0 | 0 | 2 | 0 | 1 | 0 | 1 | 0 |
| Slight | | 0 | 0 | 1 | 0 | 0 | 0 | 0 | 0 |
| Infiltrate, mononuclear cell,  spinal root/spinal nerve | |  |  |  |  |  |  |  |  |
| Total number affected | | 0 | 0 | 2 | 0 | 0 | 0 | 0 | 0 |
| Minimal | | 0 | 0 | 1 | 0 | 0 | 0 | 0 | 0 |
| Slight | | 0 | 0 | 1 | 0 | 0 | 0 | 0 | 0 |
| Aggregate, satellite glial cell | |  |  |  |  |  |  |  |  |
| Total number affected | | 0 | 0 | 0 | 0 | 0 | 0 | 0 | 0 |
| Minimal | | 0 | 0 | 0 | 0 | 0 | 0 | 0 | 0 |
| **Ganglion, thoracic dorsal root** | |  |  |  |  |  |  |  |  |
| Inflammation, mononuclear cell | |  |  |  |  |  |  |  |  |
| Total number affected | | 0 | 0 | 2 | 0 | 0 | 0 | 0 | 0 |
| Minimal | | 0 | 0 | 2 | 0 | 0 | 0 | 0 | 0 |
| Degeneration, neuron | |  |  |  |  |  |  |  |  |
| Total number affected | | 0 | 0 | 2 | 0 | 0 | 0 | 0 | 0 |
| Minimal | | 0 | 0 | 2 | 0 | 0 | 0 | 0 | 0 |
| Aggregate, satellite glial cell | |  |  |  |  |  |  |  |  |
| Total number affected | | 0 | 0 | 0 | 0 | 0 | 0 | 0 | 0 |
| Minimal | | 0 | 0 | 0 | 0 | 0 | 0 | 0 | 0 |
| **Ganglion, lumbar dorsal root** | |  |  |  |  |  |  |  |  |
| Inflammation, mononuclear cell | |  |  |  |  |  |  |  |  |
| Total number affected | | 0 | 0 | 3 | 1 | 2 | 0 | 0 | 0 |
| Minimal | | 0 | 0 | 1 | 1 | 0 | 0 | 0 | 0 |
| Slight | | 0 | 0 | 0 | 0 | 1 | 0 | 0 | 0 |
| Moderate | | 0 | 0 | 2 | 0 | 1 | 0 | 0 | 0 |
| Degeneration, neuron | |  |  |  |  |  |  |  |  |
| Total number affected | | 0 | 0 | 3 | 1 | 2 | 0 | 0 | 0 |
| Minimal | | 0 | 0 | 1 | 1 | 0 | 0 | 0 | 0 |
| Slight | | 0 | 0 | 1 | 0 | 1 | 0 | 0 | 0 |
| Moderate | | 0 | 0 | 1 | 0 | 1 | 0 | 0 | 0 |
| Degeneration, axon, spinal  root/spinal nerve | |  |  |  |  |  |  |  |  |
| Total number affected | | 1 | 0 | 3 | 0 | 2 | 0 | 2 | 0 |
| Minimal | | 1 | 0 | 1 | 0 | 0 | 0 | 2 | 0 |
| Slight | | 0 | 0 | 1 | 0 | 2 | 0 | 0 | 0 |
| Moderate | | 0 | 0 | 1 | 0 | 0 | 0 | 0 | 0 |
| Infiltrate, mononuclear cell,  spinal root/spinal nerve | |  |  |  |  |  |  |  |  |
| Total number affected | | 1 | 0 | 3 | 0 | 2 | 0 | 1 | 0 |
| Minimal | | 1 | 0 | 3 | 0 | 0 | 0 | 1 | 0 |
| Slight | | 0 | 0 | 0 | 0 | 2 | 0 | 0 | 0 |
| Aggregate, satellite glial cell | |  |  |  |  |  |  |  |  |
| Total number affected | | 0 | 0 | 0 | 0 | 0 | 1 | 0 | 0 |
| Minimal | | 0 | 0 | 0 | 0 | 0 | 1 | 0 | 0 |
| **Ganglion, sacral dorsal root** | |  |  |  |  |  |  |  |  |
| Inflammation, mononuclear cell | |  |  |  |  |  |  |  |  |
| Total number affected | | 0 | 0 | 2 | 0 | 2 | 1 | 2 | 0 |
| Minimal | | 0 | 0 | 0 | 0 | 0 | 1 | 1 | 0 |
| Slight | | 0 | 0 | 0 | 0 | 2 | 0 | 1 | 0 |
| Moderate | | 0 | 0 | 2 | 0 | 0 | 0 | 0 | 0 |
| Degeneration, neuron | |  |  |  |  |  |  |  |  |
| Total number affected | | 0 | 0 | 2 | 0 | 2 | 1 | 1 | 0 |
| Minimal | | 0 | 0 | 0 | 0 | 0 | 1 | 1 | 0 |
| Slight | | 0 | 0 | 1 | 0 | 1 | 0 | 0 | 0 |
| Moderate | | 0 | 0 | 1 | 0 | 1 | 0 | 0 | 0 |
| Hemorrhage, ganglia | |  |  |  |  |  |  |  |  |
| Total number affected | | 0 | 0 | 1 | 0 | 0 | 0 | 1 | 0 |
| Minimal | | 0 | 0 | 1 | 0 | 0 | 0 | 1 | 0 |
| Degeneration, axon, spinal  root/spinal nerve | |  |  |  |  |  |  |  |  |
| Total number affected | | 0 | 0 | 3 | 0 | 2 | 0 | 2 | 0 |
| Minimal | | 0 | 0 | 2 | 0 | 1 | 0 | 0 | 0 |
| Slight | | 0 | 0 | 0 | 0 | 1 | 0 | 1 | 0 |
| Moderate | | 0 | 0 | 1 | 0 | 0 | 0 | 1 | 0 |
| Infiltrate, mononuclear cell,  spinal root/spinal nerve | |  |  |  |  |  |  |  |  |
| Total number affected | | 0 | 0 | 2 | 0 | 1 | 0 | 0 | 0 |
| Minimal | | 0 | 0 | 2 | 0 | 1 | 0 | 0 | 0 |
| **Ganglion, trigeminal** | |  |  |  |  |  |  |  |  |
| Inflammation, mononuclear  cell | |  |  |  |  |  |  |  |  |
| Total number affected | | 0 | 0 | 0 | 0 | 0 | 0 | 0 | 1 |
| Minimal | | 0 | 0 | 0 | 0 | 0 | 0 | 0 | 1 |
| Degeneration, neuron | |  |  |  |  |  |  |  |  |
| Total number affected | | 0 | 0 | 0 | 0 | 0 | 0 | 0 | 0 |
| Minimal | | 0 | 0 | 0 | 0 | 0 | 0 | 0 | 0 |
