# Supplementary material for: Single-Dose Intrathecal Dorsal Root Ganglia Toxicity of Onasemnogene Abeparvovec in Cynomolgus Monkeys
Source: Hum Gene Ther. 2022 Jul 13;33(13-14):740–56. doi: 10.1089/hum.2021.255 (PMC9347375; doi:10.1089/hum.2021.255)
Supplement: Supplemental data [file Suppl_TableS12.docx]

**Supplemental Table 12. Summary incidence and severity of selected onasemnogene abeparvovec–related microscopic findings—dorsal root, superior cervical, and trigeminal ganglia interim necropsy at 6 weeks of observation post-intravenous dosing**

| Tissue/finding | Sex | | | | | | |
| --- | --- | --- | --- | --- | --- | --- | --- |
|  | Males | | | Females | | | |
| Dose (vg/kg) | 0 | 1.1×10^14^ | 1.1×10^14^ | 0 | 1.1×10^14^ | 1.1×10^14^ | |
| Prednisolone (mg/kg/day) | 0 | - | 1 | 0 | - | 1 | |
| Number examined | 3 | 3 | 3 | 3 | 3 | 3 | |
| **Ganglion, cervical dorsal root** |  |  |  |  |  |  | |
| Degeneration, neuron |  |  |  |  |  |  | |
| Total number affected | 0 | 3 | 2 | 0 | 2 | 1 | |
| Minimal | 0 | 2 | 2 | 0 | 1 | 1 | |
| Slight | 0 | 1 | 0 | 0 | 1 | 0 | |
| Inflammation, mononuclear cell |  |  |  |  |  |  | |
| Total number affected | 0 | 3 | 3 | 0 | 2 | 2 | |
| Minimal | 0 | 2 | 3 | 0 | 1 | 2 | |
| Slight | 0 | 1 | 0 | 0 | 1 | 0 | |
| **Ganglion, thoracic dorsal root** |  |  |  |  |  |  |  |
| Degeneration, neuron |  |  |  |  |  |  | |
| Total number affected | 0 | 1 | 1 | 0 | 1 | 1 | |
| Minimal | 0 | 1 | 1 | 0 | 1 | 1 | |
| Inflammation, mononuclear cell |  |  |  |  |  |  | |
| Total number affected | 0 | 1 | 3 | 0 | 2 | 2 | |
| Minimal | 0 | 1 | 3 | 0 | 1 | 2 | |
| Slight | 0 | 0 | 0 | 0 | 1 | 0 | |
| **Ganglion, lumbar dorsal root** |  |  |  |  |  |  | |
| Degeneration, neuron |  |  |  |  |  |  | |
| Total number affected | 0 | 1 | 3 | 1 | 2 | 3 | |
| Minimal | 0 | 0 | 1 | 1 | 1 | 2 | |
| Slight | 0 | 1 | 2 | 0 | 1 | 1 | |
| Inflammation, mononuclear cell |  |  |  |  |  |  | |
| Total number affected | 0 | 2 | 3 | 1 | 2 | 2 | |
| Minimal | 0 | 1 | 1 | 1 | 1 | 1 | |
| Slight | 0 | 1 | 2 | 0 | 1 | 1 | |
| **Ganglion, dorsal root, sacral** |  |  |  |  |  |  | |
| Degeneration, neuron |  |  |  |  |  |  | |
| Total number affected | 0 | 1 | 3 | 0 | 2 | 1 | |
| Minimal | 0 | 1 | 2 | 0 | 1 | 1 | |
| Slight | 0 | 0 | 1 | 0 | 1 | 0 | |
| Inflammation, mononuclear cell  Total number affected | 0 | 2 | 3 | 0 | 2 | 2 | |
| Minimal | 0 | 2 | 2 | 0 | 1 | 2 | |
| Slight | 0 | 0 | 1 | 0 | 1 | 0 | |
| **Ganglion, trigeminal** |  |  |  |  |  |  | |
| Degeneration, neuron |  |  |  |  |  |  | |
| Total number affected | 0 | 2 | 1 | 0 | 1 | 3 | |
| Minimal | 0 | 1 | 1 | 0 | 0 | 3 | |
| Slight | 0 | 1 | 0 | 0 | 1 | 0 | |
| Inflammation, mononuclear cell  Total number affected | 0 | 2 | 2 | 0 | 1 | 2 | |
| Minimal | 0 | 0 | 2 | 0 | 0 | 1 | |
| Slight | 0 | 2 | 0 | 0 | 1 | 1 | |
|  | | | | | | | |
