# Supplementary material for: Single-Dose Intrathecal Dorsal Root Ganglia Toxicity of Onasemnogene Abeparvovec in Cynomolgus Monkeys
Source: Hum Gene Ther. 2022 Jul 13;33(13-14):740–56. doi: 10.1089/hum.2021.255 (PMC9347375; doi:10.1089/hum.2021.255)
Supplement: Supplemental data [file Suppl_TableS14.docx]

Supplemental Table 14: Summary incidence and severity onasemnogene abeparvovec–related microscopic findings in the spinal cord and intrathecal injection site, sacral spinal cord at 6 weeks of observation post-intrathecal dosing in the 12-month GLP study

| Tissue/finding | Sex | | | | | | | |
| --- | --- | --- | --- | --- | --- | --- | --- | --- |
|  | Males | | | | Females | | | |
| Dose (vg/animal in 0.80 mL volume) | 0 | 1.2×10^13^ | 3.0×10^13^ | 6.0×10^13^ | 0 | 1.2×10^13^ | 3.0×10^13^ | 6.0×10^13^ |
| Number examined | 3 | 3 | 3 | 3 | 3 | 3 | 3 | 3 |
| **Spinal cord, cervical** |  |  |  |  |  |  |  |  |
| Degeneration, axon, dorsal  funiculus |  |  |  |  |  |  |  |  |
| Total number affected | 0 | 2 | 1 | 2 | 0 | 1 | 2 | 2 |
| Minimal | 0 | 1 | 1 | 1 | 0 | 0 | 2 | 0 |
| Slight | 0 | 1 | 0 | 1 | 0 | 1 | 0 | 2 |
| Gliosis, dorsal funiculus |  |  |  |  |  |  |  |  |
| Total number affected | 0 | 0 | 1 | 0 | 0 | 0 | 0 | 0 |
| Minimal | 0 | 0 | 1 | 0 | 0 | 0 | 0 | 0 |
| Degeneration, axon, dorsal  nerve root |  |  |  |  |  |  |  |  |
| Total number affected | 0 | 1 | 0 | 0 | 0 | 1 | 0 | 0 |
| Minimal | 0 | 0 | 0 | 0 | 0 | 1 | 0 | 0 |
| Slight | 0 | 1 | 0 | 0 | 0 | 0 | 0 | 0 |
| **Spinal cord, thoracic** |  |  |  |  |  |  |  |  |
| Degeneration, axon, dorsal  funiculus |  |  |  |  |  |  |  |  |
| Total number affected | 0 | 3 | 3 | 0 | 0 | 2 | 1 | 1 |
| Minimal | 0 | 3 | 2 | 0 | 0 | 2 | 1 | 0 |
| Slight | 0 | 0 | 1 | 0 | 0 | 0 | 0 | 1 |
| Gliosis, dorsal funiculus |  |  |  |  |  |  |  |  |
| Total number affected | 0 | 1 | 0 | 0 | 0 | 0 | 0 | 0 |
| Minimal | 0 | 1 | 0 | 0 | 0 | 0 | 0 | 0 |
| **Spinal cord, lumbar** |  |  |  |  |  |  |  |  |
| Degeneration, axon, dorsal  funiculus |  |  |  |  |  |  |  |  |
| Total number affected | 0 | 3 | 2 | 2 | 0 | 2 | 3 | 2 |
| Minimal | 0 | 2 | 1 | 2 | 0 | 1 | 3 | 1 |
| Slight | 0 | 1 | 0 | 0 | 0 | 1 | 0 | 0 |
| Moderate | 0 | 0 | 1 | 0 | 0 | 0 | 0 | 1 |
| Gliosis, dorsal funiculus |  |  |  |  |  |  |  |  |
| Total number affected | 0 | 1 | 1 | 0 | 0 | 1 | 0 | 0 |
| Minimal | 0 | 1 | 1 | 0 | 0 | 1 | 0 | 0 |
| Degeneration, axon, dorsal  nerve roots |  |  |  |  |  |  |  |  |
| Total number affected | 0 | 0 | 0 | 1 | 0 | 0 | 0 | 0 |
| Slight | 0 | 0 | 0 | 1 | 0 | 0 | 0 | 0 |
| Infiltrate, mononuclear  cell, nerve root |  |  |  |  |  |  |  |  |
| Total number affected | 0 | 0 | 0 | 1 | 0 | 1 | 0 | 0 |
| Minimal | 0 | 0 | 0 | 0 | 0 | 1 | 0 | 0 |
| Slight | 0 | 0 | 0 | 1 | 0 | 0 | 0 | 0 |
| Degeneration,  neuron/satellitosis, ventral  gray matter |  |  |  |  |  |  |  |  |
| Total number affected | 0 | 0 | 0 | 1 | 0 | 0 | 0 | 0 |
| Slight | 0 | 0 | 0 | 1 | 0 | 0 | 0 | 0 |
| **Intrathecal injection site, sacral spinal cord** |  |  |  |  |  |  |  |  |
| Degeneration, axon, dorsal  funiculus |  |  |  |  |  |  |  |  |
| Total number affected | 0 | 3 | 2 | 1 | 1 | 2 | 0 | 2 |
| Minimal | 0 | 1 | 1 | 1 | 1 | 2 | 0 | 0 |
| Slight | 0 | 2 | 0 | 0 | 0 | 0 | 0 | 1 |
| Moderate | 0 | 0 | 1 | 0 | 0 | 0 | 0 | 1 |
| Degeneration,  neuron/satellitosis,  ventral gray matter |  |  |  |  |  |  |  |  |
| Total number affected | 0 | 0 | 0 | 2 | 0 | 0 | 0 | 0 |
| Minimal | 0 | 0 | 0 | 2 | 0 | 0 | 0 | 0 |
