# Supplementary material for: Single-Dose Intrathecal Dorsal Root Ganglia Toxicity of Onasemnogene Abeparvovec in Cynomolgus Monkeys
Source: Hum Gene Ther. 2022 Jul 13;33(13-14):740–56. doi: 10.1089/hum.2021.255 (PMC9347375; doi:10.1089/hum.2021.255)
Supplement: Supplemental data [file Suppl_TableS15.docx]

Supplemental Table 15: Incidence and severity of select microscopic findings in the spinal cord at 52 weeks of observation post-intrathecal dosing in the 12-month GLP study

| Tissue/finding | Sex | | | | | | | | | | |
| --- | --- | --- | --- | --- | --- | --- | --- | --- | --- | --- | --- |
|  | Males | | | | | | Females | | | | |
| Dose (vg/animal in 0.80 mL volume) | 0 | 1.2×10^13^ | | 3.0×10^13^ | | 6.0×10^13^ | 0 | 1.2×10^13^ | 3.0×10^13^ | 6.0×10^13^ | |
| Number examined | 2 | 2 | | 2 | | 2 | 2 | 2 | 2 | 2 | |
| **Spinal cord, cervical** |  |  | |  | |  |  |  |  | |  |
| Degeneration, axon, dorsal  funiculus |  |  | |  | |  |  |  |  | |  |
| Total number affected | 0 | 1 | | 1 | | 2 | 1 | 1 | 1 | | 1 |
| Minimal | 0 | 1 | | 1 | | 2 | 1 | 1 | 1 | | 0 |
| Slight | 0 | 0 | | 0 | | 0 | 0 | 0 | 0 | | 1 |
| Gliosis, dorsal funiculus |  |  | |  | |  |  |  |  | |  |
| Total number affected | 0 | 0 | | 0 | | 0 | 0 | 0 | 0 | | 1 |
| Minimal | 0 | 0 | | 0 | | 0 | 0 | 0 | 0 | | 1 |
| **Spinal cord, thoracic** |  |  | |  | |  |  |  |  | |  |
| Degeneration, axon, dorsal  funiculus |  |  | |  | |  |  |  |  | |  |
| Total number affected | 0 | 0 | | 1 | | 1 | 0 | 0 | 1 | | 1 |
| Minimal | 0 | 0 | | 1 | | 1 | 0 | 0 | 1 | | 1 |
| Gliosis, dorsal gray matter |  |  | |  | |  |  |  |  | |  |
| Total number affected | 0 | 0 | | 0 | | 0 | 0 | 0 | 0 | | 1 |
| Minimal | 0 | 0 | | 0 | | 0 | 0 | 0 | 0 | | 1 |
| **Spinal cord, lumbar** |  |  | |  | |  |  |  |  | |  |
| Degeneration, axon, dorsal  funiculus |  |  | |  | |  |  |  |  | |  |
| Total number affected | 0 | 0 | | 1 | | 1 | 0 | 0 | 0 | | 1 |
| Minimal | 0 | 0 | | 0 | | 1 | 0 | 0 | 0 | | 0 |
| Slight | 0 | 0 | | 1 | | 0 | 0 | 0 | 0 | | 1 |
| Gliosis, ventral gray matter |  |  | |  | |  |  |  |  | |  |
| Total number affected | 0 | 0 | | 0 | | 0 | 0 | 0 | 0 | | 1 |
| Minimal | 0 | 0 | | 0 | | 0 | 0 | 0 | 0 | | 1 |
| **Intrathecal injection site,  sacral spinal cord** |  | |  |  |  | |  |  |  | |  |
| Degeneration, axon, dorsal  funiculus |  | |  |  |  | |  |  |  | |  |
| Total number affected | 0 | | 0 | 0 | 0 | | 0 | 0 | 0 | | 1 |
| Minimal | 0 | | 0 | 0 | 0 | | 0 | 0 | 0 | | 1 |
