# Supplementary material for: Single-Dose Intrathecal Dorsal Root Ganglia Toxicity of Onasemnogene Abeparvovec in Cynomolgus Monkeys
Source: Hum Gene Ther. 2022 Jul 13;33(13-14):740–56. doi: 10.1089/hum.2021.255 (PMC9347375; doi:10.1089/hum.2021.255)
Supplement: Supplemental data [file Suppl_TableS16.docx]

**Supplemental Table 16.** **Summary incidence and severity of onasemnogene abeparvovec–related microscopic findings in the peripheral nerves at 6 weeks of observation post-intrathecal dosing** **in the 12-month GLP study**

| Tissue/finding | Sex | | | | | | | |
| --- | --- | --- | --- | --- | --- | --- | --- | --- |
|  | Males | | | | Females | | | |
| Dose (vg/animal in 0.80 mL volume) | 0 | 1.2×10^13^ | 3.0×10^13^ | 6.0×10^13^ | 0 | 1.2×10^13^ | 3.0×10^13^ | 6.0×10^13^ |
| Number examined^a^ | 3 | 3 | 3 | 3 | 3 | 3 | 3 | 3 |
| **Nerve, fibular** |  |  |  |  |  |  |  |  |
| Degeneration, axon |  |  |  |  |  |  |  |  |
| Total number affected | 0 | 2 | 0 | 0 | 0 | 1 | 0 | 3 |
| Minimal | 0 | 2 | 0 | 0 | 0 | 1 | 0 | 2 |
| Slight | 0 | 0 | 0 | 0 | 0 | 0 | 0 | 1 |
| **Nerve, tibial** |  |  |  |  |  |  |  |  |
| Degeneration, axon |  |  |  |  |  |  |  |  |
| Total number affected | 0 | 3 | 0 | 2 | 1 | 2 | 0 | 3 |
| Minimal | 0 | 2 | 0 | 2 | 1 | 1 | 0 | 3 |
| Slight | 0 | 1 | 0 | 0 | 0 | 1 | 0 | 0 |
| **Nerve, sural** |  |  |  |  |  |  |  |  |
| Number examined | 3 | 2 | 3 | 3 | 3 | 3 | 3 | 3 |
| Degeneration, axon |  |  |  |  |  |  |  |  |
| Total number affected | 0 | 2 | 0 | 0 | 0 | 1 | 0 | 3 |
| Minimal | 0 | 2 | 0 | 0 | 0 | 1 | 0 | 3 |
| **Nerve, sciatic** |  |  |  |  |  |  |  |  |
| Degeneration, axon |  |  |  |  |  |  |  |  |
| Total number affected | 1 | 3 | 1 | 1 | 0 | 2 | 0 | 3 |
| Minimal | 1 | 2 | 1 | 1 | 0 | 2 | 0 | 3 |
| Slight | 0 | 1 | 0 | 0 | 0 | 0 | 0 | 0 |
| ^a^Number examined unless otherwise stated. | | | | | | | | |
