# Supplementary material for: Single-Dose Intrathecal Dorsal Root Ganglia Toxicity of Onasemnogene Abeparvovec in Cynomolgus Monkeys
Source: Hum Gene Ther. 2022 Jul 13;33(13-14):740–56. doi: 10.1089/hum.2021.255 (PMC9347375; doi:10.1089/hum.2021.255)
Supplement: Supplemental data [file Suppl_FigS3.docx]

**Supplemental Figure 3. *In situ* hybridization** **(ISH) analysis of dorsal root ganglion (DRG).** Vector expression was detected in sensory neurons of DRG following intrathecal administration of onasemnogene abeparvovec (6.0×10^13^ vg/animal) at interim necropsy, 6 weeks post-dose. ISH of DRG using an onasemnogene abeparvovec antisense (A) and sense (B) probes. Magnification: 20×.

**
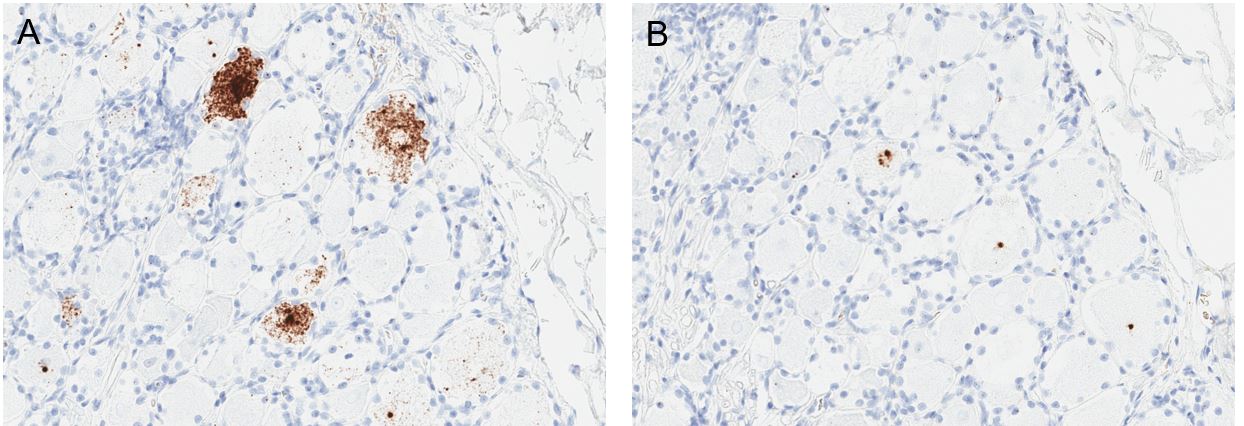
**
