# Supplementary material for: Single-Dose Intrathecal Dorsal Root Ganglia Toxicity of Onasemnogene Abeparvovec in Cynomolgus Monkeys
Source: Hum Gene Ther. 2022 Jul 13;33(13-14):740–56. doi: 10.1089/hum.2021.255 (PMC9347375; doi:10.1089/hum.2021.255)
Supplement: Supplemental data [file Suppl_Appendix.docx]

**SUPPLEMENTAL APPENDIX**

**SUPPLEMENTAL METHODS**

**Animals**

For the 12-month intrathecal study and 6-month intravenous study, cynomolgus monkeys of Asian origin were received from Worldwide Primates, Inc. (Miami, FL, USA). For the 13-week study, cynomolgus monkeys of Mauritius origin were received from the Noveprim Group (Mahebourg, Mauritius).

**Intravenous study design**

The study design included three dose groups of cynomolgus monkeys (vehicle control, onasemnogene abeparvovec alone, and onasemnogene abeparvovec in combination with the immunosuppressant [prednisolone]). Onasemnogene abeparvovec and vehicle control article were administered once on Day 1 of the dosing phase by intravenous infusion via a saphenous vein for approximately 20 minutes using a calibrated external pumping device. Animals in each dose group were also administered placebo (reverse osmosis water) or prednisolone (3 mg/mL) at a dose volume of 0.33 mL/kg by oral administration once daily from the day prior to dose administration through Day 29 of the dosing phase and on Days 31, 33, 35, 37, 39, and 41 of the dosing phase. In summary, one group (n=6 animals/sex) of cynomolgus monkeys was administered vehicle control article and placebo and served as controls (Group 1); a second group (n=6 animals/sex) was administered 1.1×10^14^ vg/kg onasemnogene abeparvovec (the approved clinical dose) and placebo (Group 2); and a third group (n=6 animals/sex) was administered 1.1×10^14^ vg/kg and prednisolone (Group 3).

**Pre-study serum anti-AAV9 antibody titers**

A Meso Scale Discovery homogenous bridging immunoassay was used to determine anti-AAV9 antibody titers. The samples from the 12-month GLP and the 6-month intravenous GLP studies were analyzed in the same bioanalytical laboratory. This assay had a minimal required dilution (MRD) of 20 and used two-fold dilution steps. The titer was reported as the reciprocal of the lowest dilution that yielded a signal greater than, or equal to, the plate-specific cut point.

The samples from the 13-week mechanistic study were analyzed at a different laboratory using a MRD of 40 and six-fold dilution steps. The titer was calculated as the dilution factor at the intercept with the cut point generated by the dilution just above and just below the

cut point and reported as log_10_ of this dilution.

**Onasemnogene abeparvovec DNA concentrations in DRG**

Onasemnogene abeparvovec DNA concentrations were determined using a droplet digital polymerase chain reaction (ddPCR) assay. The primers and probe used for this assay were designed to distinguish between onasemnogene abeparvovec DNA and endogenous *SMN* DNA. Data are reported as vg copies/diploid genome.

***In situ* hybridization**

*In situ* hybridization (ISH) to detect onasemnogene abeparvovec antisense (AS) and sense (S) sequences as well as the *macaca mulatta peptidyl-prolyl cis-trans isomerase B* (*Mmu-PPIB*) gene (a constitutively expressed positive control and tissue quality control) and the *dihydrodipicolinate reductase* (*DAPB*) gene (a negative control gene) was performed on tissue sections at 6 weeks post-dose (6.0×10^13^ vg/animal) using reagents and equipment supplied by Advanced Cell Diagnostics (ACDBio; Hayward, CA) and Ventana Medical Systems (Roche, Tucson, AZ). The following ISH RNAscope^®^ probes were designed by ACDBio: RNAscope^®^ 2.5 VS Probe Mmu-PPIB (Cat. #457719), RNAscope^®^ 2.5 VS Probe DAPB (Cat. #312039), RNAscope^®^ 2.5 VS Probe AAV phSMN AS (Cat. #827159), and RNAscope^®^ 2.5 VS Probe AAV phSMN S (Cat. #831649). AS and S probes targeted vector-unique sequences within the transgene and polyA regions and did not produce signal in undosed control animal tissues. The AS probe detects vector RNA and DNA. The S probe detects primarily vector DNA but also may detect small amounts of vector RNA produced through promoter activity off the right-side inverted terminal repeat. The nature of the sequences detected can be further elucidated by pretreatment of tissue sections with DNase and RNase. Appropriate positive *Mmu-PPIB* and negative *DAPB* controls were included to ensure mRNA quality and specificity, respectively. The hybridization method followed protocols established by ACDBio and Ventana systems using a 3,3’-Diaminobenzidine chromogen. Briefly, 5 μm sections were baked at 60°C for 60 minutes and used for hybridization. The deparaffinization and rehydration protocol was performed using a Sakura Tissue-Tek DR5 stainer with the following steps: three times xylene for 3 minutes each; two times 100% alcohol for 3 minutes; air dried for 5 minutes. Off-line manual pretreatment in 1× retrieval buffer at 98–104°C for 15 minutes. Optimization was performed by first evaluating *PPIB* and *DAPB* hybridization signal and subsequently using the same conditions for all slides. Following pretreatment, the slides were transferred to a Ventana Ultra autostainer to complete the ISH procedure including protease pretreatment, hybridization at 43°C for 2 hours followed by amplification, and detection with horseradish peroxidase and hematoxylin counter stain.

**Biomarker assays**For neurofilament light chain (NfL) analysis in the 12-month study, K2-EDTA serum samples collected from a femoral vein, and cerebrospinal fluid (CSF) samples collected from the lumbar region, pre-dose, and cisterna magna, on Days 22, 43, 78, and 162 of the dosing phase and on days of euthanasia, were received and stored frozen at –80°C. Serum and CSF samples were thawed and analyzed at four- and 100-fold dilution (or eight- and 200-fold when repeat analysis was required for values above the limit of detection), respectively, for NfL using the qualified Simoa™ NF-light^®^ immunoassay according to the manufacturer’s instructions (Cat. #103186; Quanterix, Lexington, MA). Quantification was performed on the Quanterix Simoa SR-X Analyzer, and data were analyzed using SoftMax Pro v5.4.1 (Molecular Devices, Sunnyvale, CA).

For NfL analysis in the 13-week mechanistic study, K2-EDTA plasma samples collected from the vena cephalica antebrachii or vena saphena once in the pre-dose phase and on Day 7 and end of Weeks 2, 5, and 13 of the dosing phase were received and stored frozen at ‒70°C or below. Plasma and CSF samples were thawed and analyzed at a four- and 100-fold dilution, respectively, for NfL using the qualified Simoa™ Neurology 4 Plex B immunoassay (Cat. #103345, Quanterix) according to the manufacturer’s instructions. Quantification was performed on the Quanterix Simoa SR-X Analyzer, and data were analyzed using SoftMax Pro v5.4.1 (Molecular Devices, Sunnyvale, CA).

For NfL analysis in the GLP-compliant 6-month intravenous study, K2-EDTA plasma samples collected from the femoral vein once in the pre-dose phase and on Days 7, 12, 33, and 89 of the dosing phase were received and stored frozen at ‒80°C or below. Plasma samples were thawed and analyzed at a four- and 100-fold dilution, respectively, for NfL using the qualified Simoa™ Neurology 4 Plex B immunoassay (Cat. #103345, Quanterix) according to the manufacturer’s instructions. Quantification was performed on the Quanterix Simoa SR-X Analyzer, and data were analyzed using SoftMax Pro v5.4.1 (Molecular Devices, Sunnyvale, CA).

**Neurologic Examinations**

In the 13-week intrathecal study, neurologic examinations were performed on all animals twice during the pre-dose phase, on Day 1 (pre-dose) and approximately 4 hours after dosing; Days 3 and 10; and every 2 weeks (Weeks 5, 7, 9, 11, and 13) following the interim necropsy. Neurologic examinations were conducted on unsedated animals and included general sensorimotor aspects, cranial reflexes (pupillary and orbicularis oculi), spinal reflexes (patellar and anal), and foot grip reflex.

12-month intrathecal GLP study: neurologic examinations were conducted on unanesthetized animals twice during the pre-dose phase; approximately 4 and 24 hours post-dose on Day 1; and once during Weeks 2, 3, 4, and 6 of the dosing phase. Post-dose examinations were based on the dosing completion time for each individual animal. Neurologic examinations included locomotor activity, behavioral changes, coordination, posture, tremors, convulsion, pupil evaluations, and reflex assessments.

GLP-compliant 6-month intravenous study: neurologic examinations were conducted on unanesthetized animals by a veterinarian during the pre-dose phase; on Day 1 of the dosing phase at approximately 4 hours post-dose; Day 2 at approximately 24 hours post-dose; and during Weeks 3, 6, 13, and 25 of the dosing phase. The head and eyes were examined for unusual orientation and movements. Reflexes were tested (including menace, pupillary, palpebral, limb flexor, and patellar) and muscle tone of unrestrained limbs was evaluated. Neurologic examinations included locomotor activity, behavioral changes, coordination, posture, tremors, convulsion, pupil evaluations and reflex assessments. Special attention was directed at assessment of peripheral sensory nervous system function.

**Neuroelectrophysiologic evaluations (neurography)**
In the 13-week intrathecal study, peripheral nerve function (sensory, sural lateral dorsal cutaneous branch nerve, superficial fibular sensory nerve and saphenous nerve; motor, tibial branches of the sciatic nerve) following intrathecal onasemnogene abeparvovec was assessed by neurography at 2- and 13-weeks post-dose (3×10^13^ vg/animal) for all animals once during the pre-dose phase and prior to euthanasia (one day before interim and 4 days before terminal necropsy). Animals were anesthetized with ketamine and medetomidine. Atipamezole was used as an antidote at the end of investigations. The duration of the neurography was approximately 20 minutes.

Signal collection and post-collection data analyses were performed with a calibrated, clinically certified clinical electromyography machine. Natus subdermal sterile platinum needle electrodes (<12 mm/0.3 mm or equivalent) were used for recording. A pediatric stimulator was used for eliciting evoked potentials in primates.

Nerve conduction was assessed for up to three sensory nerves of the lower limb with roots in the lumbosacral plexus (i.e., branches of the femoral nerve or the sural nerve). Motor nerve function was assessed by eliciting compound muscle action potentials (CMAPs or M-waves) from distal muscles innervated by the sciatic/tibial nerve, following orthodromic stimulation of the nerve at two locations along its course. F-wave was tested for at least one motor pathway on the lower limb. F-waves were considered confirmatory only.

Locations for stimulation and recording were consistently located relative to anatomical landmarks (e.g., lateral or medial hock, sciatic notch, interdigital space), except where anastomoses were present requiring a different stimulation and recording montage. Antidromic (for sensory nerve conduction studies) or orthodromic (for motor nerve conduction studies) nerve action potentials (relative to physiologic conduction in the respective nerves, per clinical conventions) were elicited in sensory nerves with depolarizing stimulation.

The recording electrodes (i.e., two subcutaneous needle electrodes) were positioned over the nerve or the muscle, respectively, and the nerve was stimulated proximally from the recording site with a pediatric stimulator. Two locations were stimulated for motor nerves to subtract the contribution of the neuromuscular junction. The stimulus strength was progressively increased until a maximal amplitude response was evoked (i.e., a sensory nerve action potentials or SNAP), as visualized on the machine’s screen. Approximately 10 supramaximal stimuli were averaged for each sensory nerve, at the maximum amplitude elicited. The distance from the recording site to the stimulation cathode was documented, and the conduction velocity was calculated by preset software protocols using the onset latency of the response and the distance.

**SUPPLEMENTAL RESULTS**

**Onasemnogene abeparvovec biodistribution and immunogenicity following intravenous administration**

After intravenous administration, onasemnogene abeparvovec distributed to all tissues was analyzed, including the spinal cord and dorsal root ganglion ([DRG]; data not shown). A rapid and robust humoral immune response to the adeno-associated virus (AAV) capsid was observed in both serum and CSF (data not shown).

**Peripheral nerve function following intravenous onasemnogene abeparvovec**

Neuroelectrophysiologic evaluations (neurography) assessing peripheral nerve

function approximately 5 and 26 weeks after a single intravenous infusion of onasemnogene abeparvovec to juvenile cynomolgus monkeys with or without prednisolone demonstrated peripheral sensory nerve conduction function remaining within normal physiologic range, with no decreases from control in the sural, peroneal, and saphenous sensory nerves.

**Case studies of AAV-mediated DRG toxicity**

Although AAV-mediated DRG toxicities were observed in nonclinical studies, the clinical significance of these findings remains uncertain. Currently, there are two reports of neuronal loss within the DRG of patients who have been treated with AAV gene therapies and have subsequently died, potentially as a result of the underlying disease.

The first case occurred in a patient who was enrolled in a clinical study for familial amyotrophic lateral sclerosis who received a 4.2×10^14^ vg intrathecal dose of AAV-miR-SOD1, an AAVrh10 containing an anti-superoxide dismutase 1 microRNA.^1^ The patient died at 15.6 months post-dose. An autopsy reported loss of motor neurons in parts of the spinal cord and neuronal loss in the DRG.

The second case occurred in a patient who was enrolled in a clinical trial (NCT02362438) that evaluated an AAV9 gene therapy for the treatment of giant axonal neuropathy. At the NCATS/NIH Workshop on Systemic Immunogenicity Considerations for AAV-Mediated Gene Therapy, the death of a subject (at 8 months post-dose) with advanced disease who received an intrathecal dose of 3.5×10^13^ vg (the lowest dose tested) was presented. An autopsy revealed severe neuronal loss in the absence of inflammation or clinical signs or symptoms of DRG toxicity.^2^ Younger patients with earlier disease states who were administered the same or greater doses of the gene therapy had not developed signs of DRG toxicity. The integrity of the DRG was lost early in the disease process. This case study has yet to be published.

Although these two case studies are sometimes presented as evidence of DRG pathology as a consequence of administration of an AAV gene therapy to patients, the DRG pathology inherent in these disease states confounds the interpretation of the data from these studies.
